# Higher Yield of Common Buckwheat (*Fagopyrum esculentum* Moench) as a Result of Seed Treatment with Gamma Radiation

**DOI:** 10.3390/ijms26104587

**Published:** 2025-05-10

**Authors:** Agnieszka Płażek, Przemysław Kopeć, Barbara Mickowska, Marek Szklarczyk, Wojciech Wesołowski, Anna Szczerba, Marta Hornyák, Beata Biesaga, Damian Kabat

**Affiliations:** 1 Department of Plant Breeding, Physiology and Seed Sciences, Faculty of Agriculture and Economics, University of Agriculture in Kraków, Podłużna 3, 30-239 Kraków, Poland; anna.szczerba@urk.edu.pl; 2 The Franciszek Górski Institute of Plant Physiology, Polish Academy of Sciences, Niezapominajek 21, 30-239 Kraków, Poland; p.kopec@ifr-pan.edu.pl; 3 Department of Plant Products Technology and Food Hygiene, Faculty of Food Technology, University of Agriculture in Kraków, Balicka 122, 30-149 Kraków, Poland; barbara.mickowska@urk.edu.pl; 4 Department of Plant Biology and Biotechnology, Faculty of Biotechnology and Horticulture, University of Agriculture in Kraków, 29 Listopada 54, 31-425 Kraków, Poland; marek.szklarczyk@urk.edu.pl (M.S.); wojtek.wes@gmail.com (W.W.); 5 W. Szafer Institute of Botany, Polish Academy of Sciences, Lubicz 46, 31-512 Kraków, Poland; m.hornyak@botany.pl; 6 Maria Sklodowska-Curie National Research Institute of Oncology, Kraków Branch, Garncarska 11, 31-115 Kraków, Poland; beata.biesaga@onkologia.krakow.nio.gov.pl (B.B.); damian.kabat@krakow.nio.gov.pl (D.K.)

**Keywords:** amino acid content, common buckwheat, gamma radiation, genotyping by sequencing, seed yield

## Abstract

Common buckwheat (*Fagopyrum esculentum* Moench), a valuable plant, is characterized by a highly unstable seed yield. The objective of the present study was to ascertain whether seed treatment with gamma radiation of 30 or 40 Gy would enhance yield and the content of amino acids. Plants obtained from irradiated seeds were named M0 generation. Subsequently, the mutants were subjected to cross-pollination under isolation conditions within groups, contingent upon the radiation dose, to yield the M1, M2, and M3 generations. To estimate the extent of mutation changes, genotyping by sequencing analysis was performed on selected M0 plants. Each year, the selection of plants was based on their seed yield. The amino acid composition in the seeds of the M2 and M3 generations was determined. The number of unique heterozygote variants identified for the 40 Gy plants was found to be less than that observed in remaining plants. The M0 and M1 plants of 40 Gy group exhibited an augmented seed yield; however, this characteristic did not reoccur in the M2 generation. In the M2, the control seeds exhibited the highest amino acid content compared to the mutants. In the M3 generation, only one accession of the 40 Gy group demonstrated higher amino acid content than the other plants. The findings suggest that irradiating seeds with 40 Gy can enhance seed yield and amino acid content.

## 1. Introduction

Contemporary civilization diseases encompass food allergies to numerous substances, including proteins and gluten, which can result in skin diseases, asthma, and intestinal disorders [1,2]. Consequently, there is an imperative to identify novel cultivars of crops that exhibit not only enhanced resilience to environmental stresses but also serve as alternative food sources devoid of allergenic components and abundant in vitamins, minerals, and antioxidants.

Common buckwheat (*Fagopyrum esculentum* Moench) is a plant from the *Polygonaceae* family that is primarily cultivated for its seeds, which have a favorable chemical composition. The seeds contain a substantial amount of starch, minerals, vitamins, rutin, dietary fibers, and a distinctive amino acid composition, particularly abundant in lysine and arginine. Notably, buckwheat is a notable source of antioxidants and is devoid of gluten [3,4,5,6]. Buckwheat seeds contain high amounts of macro-elements, including phosphorus, potassium, calcium, and magnesium, as well as valuable micro-elements, particularly iron and zinc [7]. Moreover, buckwheat flour has been found to contain a higher protein content in comparison to rice, wheat, sorghum, and maize [8]. Additionally, buckwheat has been shown to have medicinal properties due to its high content of phenolic compounds, which have antioxidant properties [9,10,11]. Furthermore, buckwheat flour is utilized in the production of pasta and groats, while the straw serves as animal feed [12]. Additionally, buckwheat has been identified as a valuable melliferous plant [13,14,15]. This plant species offers several advantages, including its capacity to protect against soil erosion, its ability to absorb high levels of nitrogen and phosphorus from the soil, and its resistance to pests and diseases. However, despite these numerous advantages, its agricultural potential remains underutilized due to the low and unstable yield of seeds. The low yield of buckwheat seeds is primarily attributed to its specific flowering biology and high sensitivity to environmental factors. Embryological development exhibits a high degree of susceptibility to disturbances, which are genetically determined [12,16,17]. Buckwheat exhibits self-incompatibility due to the presence of heterostyly, resulting in pin and thrum flowers with disparate pistil and stamen lengths. Fertilization occurs exclusively following cross-pollination between distinct types of flowers—a process that must transpire within a single day [18]. Common buckwheat also exhibits high levels of female sterility, yet its pollen possesses a viability rate exceeding 90% [16]. Embryo death can occur due to inadequate assimilate distribution during prolonged flowering and simultaneous seed filling. Even in cases where pollination occurs, a considerable proportion of buckwheat seeds are found to be empty, suggesting the occurrence of embryo death due to starvation. This phenomenon may be attributed to the intense competition for assimilates triggered by buckwheat’s indeterminate flowering throughout the growing season [19,20].

Contemporary Polish buckwheat breeding is undergoing a regression, which may be surprising considering the aforementioned benefits of this plant in terms of pro-health and phytosanitary value. One potential explanation for this phenomenon may be attributed to the a very poor genomic pool and the lack of plants with greater fertility and tolerance to stresses such as drought and elevated temperature. The augmentation of plant genetic diversity can be facilitated through the utilization of mutagenic factors. A substantial aim of research has been dedicated to the subject of obtaining new forms of buckwheat through mutation, as evidenced by numerous studies documented in the extant literature [21].

According to Micke et al. [22], chemical mutagens are less effective than physical agent radiation. Mathur’s research yielded notable outcomes. He exposed seeds of *F. esculentum* and *F. tataricum* to gamma rays in the range of 5–40 kR, achieving optimal results for seeds exposed to 15 kR. The regenerates exhibited the shortest time to developing the first leaf, the highest number of lateral shoots, the greatest plant height, the highest number of seeds produced, and the greatest weight in comparison with the plants obtained from seeds exposed to other doses. It is a well-documented consequence of irradiating seeds that the resultant plants often exhibit a dwarf stature. Dwarf buckwheat forms have been observed to exhibit limited growth but enhanced leaf and root development. Furthermore, mutants displayed short internodes, increased floral production, enhanced resistance to environmental conditions, and elevated seed yield compared to the control plants [23]. Some authors observed that the number of first-class branches changed significantly with a growing intensity of gamma radiation (according to Mathur [23]). Morishita et al. [17] conducted a cross between dwarf mutants of *F. tataricum*, which were obtained through irradiation with non-mutated plants. The ratio of obtained F1 plants was 1:3, indicating that this trait is governed by a single recessive gene. Mittal et al. [21] induced a mutation of *F. tataricum* buckwheat using gamma radiation and EMS (ethyl methane sulphonate). The researchers found that the mutation efficiency of gamma radiation was higher than that of EMS, and they observed a synergistic effect of both methods. Concurrently, Kurowska et al. [24] attained the maximum mutation frequency following barley seed treatment with 1.5 mM MNU or 210 Gy. It is noteworthy that the mutation effect was evaluated in the M2 generation. Gregori and Kreft [25] conducted investigations on common buckwheat mutants characterized with low content of starch. This observation serves as a foundation for the subsequent analysis of the chemical composition of buckwheat seeds derived from these mutated plants.

The research hypothesis of the study was that gamma-irradiated buckwheat mutants could exhibit increased seed yield. The objective of the study was to ascertain whether seed treatment with gamma radiation of 30 or 40 Gy could enhance seed yield (number and weight of ripe seeds from one plant with simultaneously lower number of empty seeds) and increase the amount of amino acids. The study was carried out on the common buckwheat cv. ‘Korona’, which was the subject of our earlier studies (previous accession PA15) and has a more stable seed yield compared to other Polish accessions [16,20]. The radiation doses applied were selected on the basis of pilot experiments. In these studies, we used doses of 10, 20, 30, 40 and 100 Gy, but the best results were obtained with 30 and 40 Gy. Higher doses reduced the percentage of germinated seeds to a few percent. Polish buckwheat breeding does not have a sufficient genetic pool of common buckwheat that could be the starting material for creative breeding aimed at producing new cultivars with higher, more stable yields. Gamma radiation can induce new positive traits, but can also negatively affect the chemical composition of the seeds. Our aim, therefore, was to investigate whether increasing seed yield through seed irradiation would at the same time deteriorate the composition of amino acids, the amount of which distinguishes buckwheat compared to cereals. Research in this respect has not yet been conducted.

## 2. Results

### 2.1. Seed Germination and Leaf Area

The control seeds, which were not exposed to gamma radiation, exhibited 100% germination. However, the application of 30 Gy led to a marginal reduction in the percentage of germinated seeds, with a decrease to 95%, while 40 Gy exhibited a more pronounced effect, reducing the percentage to 49%. The study observed a significant influence of radiation on the variation in leaf area, as measured five weeks after seed sowing. Plants cultivated from seeds that had undergone 30 Gy exhibited significantly larger leaves in comparison to those of plants exposed to 40 Gy. The leaves of 40 Gy group differed from those of the control plants (see Table 1 for details). No other anomalies in plant morphology and development were observed because of the irradiation.

### 2.2. Biometric Parameters

#### 2.2.1. M0 Generation

**Figure 1:**
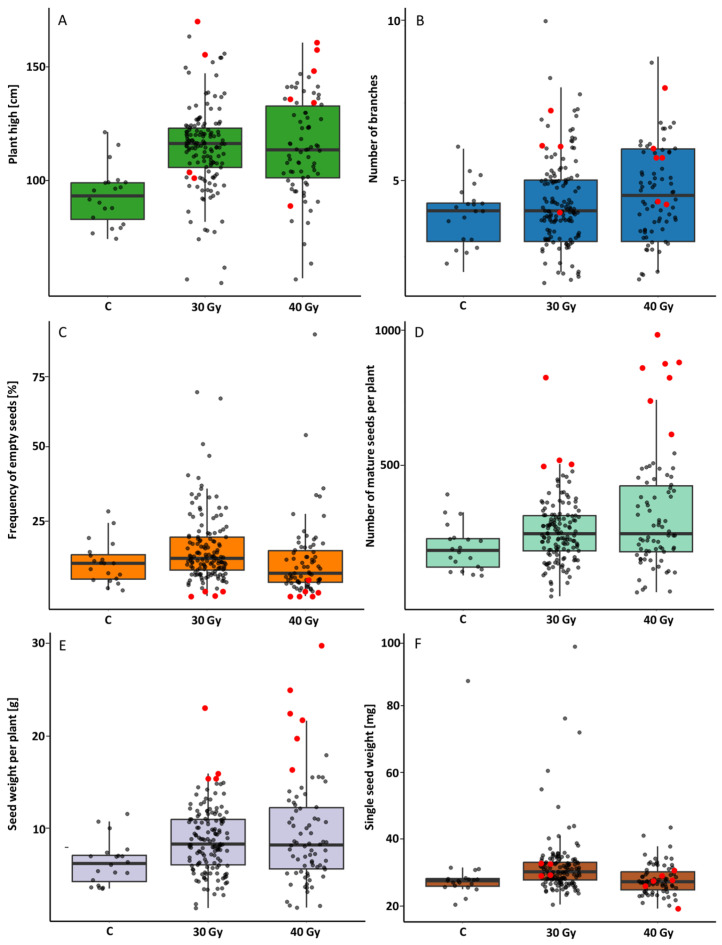
The effect of gamma irradiation of common buckwheat seeds at two doses of 30 and 40 Gy on the height of plants (**A**), the number of branches (**B**), the percentage of empty seeds (**C**), the number of mature seeds (**D**), the weight of mature seeds (**E**) and the weight of one seed of plants (**F**) in the initial generation M0. The data have been presented in Tukey’s box plots with a mark of raw data. Boxes represent the first and third quartiles with a median line; whiskers connect the values within 1.5 times the interquartile range. Accessions whose progeny were sown for analysis the following year are marked in red.

Analyses were conducted on a total of 20 control plants, 142 plants cultivated from seeds that had been exposed to 30 Gy (hereafter referred to as the 30 Gy group), and 75 plants cultivated from seeds that had been exposed to 40 Gy (hereafter referred to as the 40 Gy group). The varied number of plants in the 30 Gy and 40 Gy groups can be attributed to the varying levels of germination capacity exhibited by irradiated seeds. The numerical data of estimated parameters were presented in the Supplementary Table S1. The mutant plants exhibited significantly increased growth relative to the control plants. However, the difference in height between the lowest and highest plants was considerably greater in the mutant plants than in the control plants, at 120 cm for the 30 Gy group, 180 cm for the 40 Gy group, and 49 cm for the control plants (Figure 1A). Furthermore, a greater number of first-class branches was observed in plants cultivated from irradiated seeds in comparison to the control plants (Figure 1B). The range of values between the minimum and maximum number of first-class branches was eight in the 30 Gy plant group and seven in the 40 Gy group, which was significantly greater than in the control plants (four branches). The highest differences between the minimum and maximum fresh weight (FW) of stems were observed in the 40 Gy group, while the differences between the minimum and maximum dry weight (DW) were highest in the 30 Gy group (Supplementary Table S1). Furthermore, mutant plants produced a very large number of empty seeds (Figure 1C), although plants that did not produce any empty seeds produced very small numbers of mature seeds (Figure 1D). The maximum number of seeds was produced by the plant from the 40 Gy group (1027 seeds). Furthermore, plants from the 30 Gy group exhibited a higher level of seed production compared to the control plants, with a maximum of 861 seeds recorded. This observation was further corroborated by the mass of seeds obtained from each plant (Figure 1E). Of particular interest was the observation that, among the control plants, the maximum mass of a single seed was obtained at 0.0892 g in the plant from the 30 Gy group, while in the plants from the 40 Gy group, the maximum mass of a single seed was only 0.0441 g (Figure 1F). Conversely, in the latter group of plants, the dispersion in the mass of a single seed was the least significant when compared to that of the control and the 30 Gy group.

Accessions that were distinguished by the highest number and weight of seeds obtained from a single plant, the lowest percentage of empty seeds, and a relatively high weight of single seeds were selected for further studies. Seeds from the following accessions (progeny named M1) were collected and sown in the following year of study: 30/9, 30/33, 30/46, 30/131, 40/11, 40/18, 40/20, 40/23, 40/27, 40/35. The numerals 30 and 40 refer to the radiation dose, with the subsequent number denoting the individual plant from which seeds were obtained.

#### 2.2.2. M1 Generation

**Figure 2:**
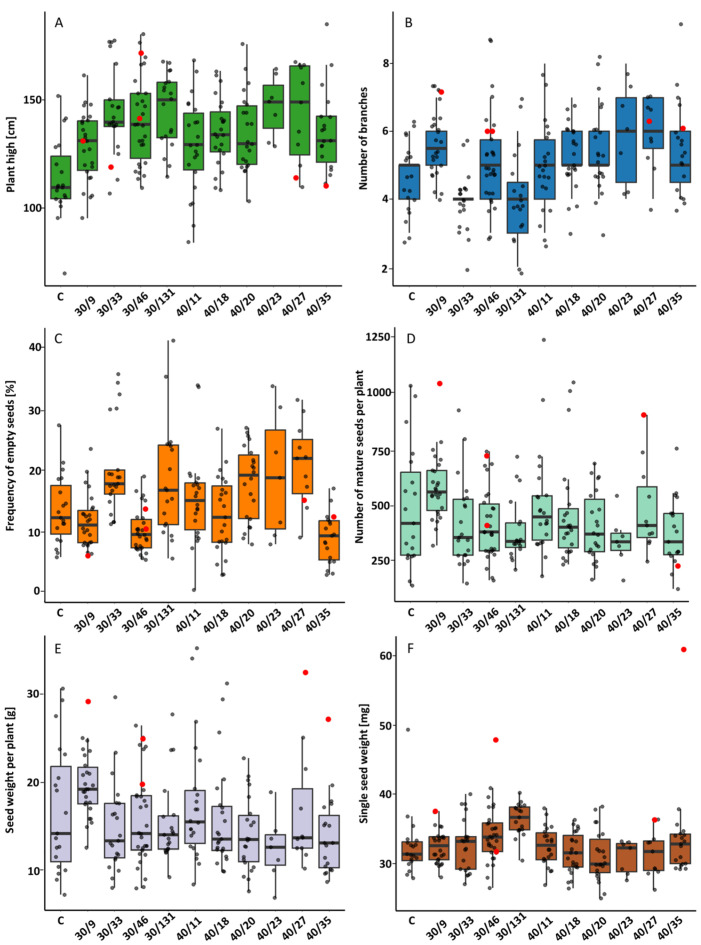
The effect of gamma irradiation of common buckwheat seeds at two doses of 30 and 40 Gy on the height of plants (**A**), the number of branches (**B**), the percentage of empty seeds (**C**), the number of mature seeds (**D**), the weight of mature seeds (**E**), and the weight of one seed (**F**) of chosen accessions of the M1 generation (**F**). The data have been presented in Tukey’s box plots with a mark of raw data. Boxes represent the first and third quartiles with a median line; whiskers connect the values within 1.5 times the interquartile range. Finally, red coloration was used to denote accessions whose progeny were sown for analysis in the following year. The numerals 30 and 40 refer to the radiation dose, with the subsequent number denoting the individual plant from which seeds were obtained.

The results of the study’s various parameters are presented in Supplementary Table S2 and Figure 2. The height of the plants was found to be significantly higher in the progeny of mutant plants (Figure 2A).

The lowest observed standard deviation for this parameter was recorded in plants from accession 40/23 (38 cm), while the highest was observed in accession 40/11 (89 cm), although this was comparable to that observed in the control plants. Within each treatment, considerable variations in the given parameters were observed. The number of first-class branches exhibited significant variation within the progeny of the 30 and 40 Gy groups, surpassing that observed in the control plants (Figure 2B). Conversely, the lowest number of empty seeds was observed in accessions 30/46 and 40/35, concurrently exhibiting the narrowest dispersion for this parameter (Figure 2C). The maximum seed number per plant was obtained from accessions 40/11 (1239 seeds), 40/18 (1047 seeds), 30/9 (1040 seeds) and from the control (1032 seeds) (Figure 2D). Conversely, accession 40/23 yielded the lowest number of seeds. The most pronounced dispersion in this parameter was observed in the control plants, and the least pronounced among the progeny of the lowest yielding accession 40/23. The maximum seed weight was obtained from accessions 40/11 (35.80 g) and 40/18 (31.39 g), yet the largest difference between the minimum and maximum seed weight was noted in accession 40/11 (29.78 g), while the smallest scatter was determined in accession 40/23 (13.29 g) (Figure 2E). The mean difference in seed weight between the control plants was 25.99 g. The most uniform single seed weight was observed in accession 40/23, with a maximum difference of 0.0057 g between the minimum and maximum weights. The highest single seed weight was recorded in the progeny of M1 from 40/35, at 0.0615 g.

#### 2.2.3. M2 Generation

**Figure 3:**
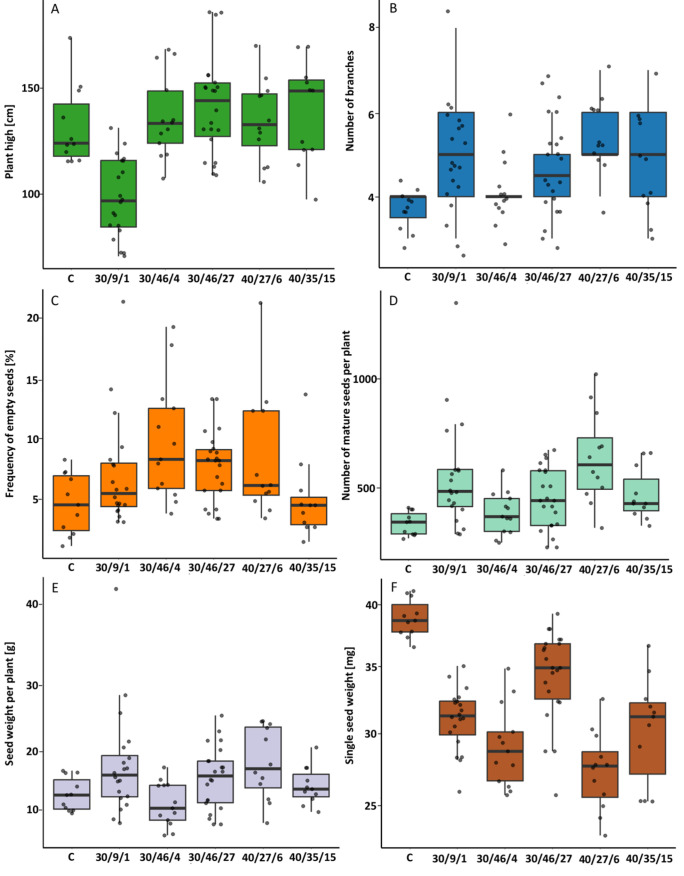
The effect of gamma irradiation of common buckwheat seeds at two doses of 30 and 40 Gy on the height of plants (**A**), the number of branches (**B**), the percentage of empty seeds (**C**), the number of mature seeds (**D**), the weight of mature seeds (**E**), and the weight of one seed (**F**) of chosen accessions of the M2 generation. The data have been presented in the form of Tukey’s box plots, with the raw data points indicated by a marker. Boxes represent the first and third quartiles with a median line; whiskers connect the values within 1.5 times the interquartile range. The numerals 30 and 40 refer to the radiation dose, with the subsequent number denoting the individual plant from which seeds were obtained.

Analyses were conducted on the progeny of the following accessions: 30/9/1, 30/46/4, 30/46/27, 40/27/6 and 40/35/15. The numeric data of biometric parameters are presented in Supplementary Table S3. The progeny M2 of the 30/9/1 accession exhibited lower values than the plants of M1—30/9, and significantly lower values than the control plants and other accessions under study (Figure 3A).

Mutant heights of the M2 progeny were more uniform than those observed in the M1 generation, although individual plants showed significant variation from the other plants. The plant with the highest recorded height was obtained from accession 30/46/27, measuring 188 cm. Furthermore, all mutants exhibited an increased number of first-class branches in comparison to the control plants, with the exception of 30/46/4 (Figure 3B). Furthermore, a positive phenomenon was observed, with a significantly smaller number of empty seeds being produced by mutants (Figure 3C). The mutant accession 40/35/15 exhibited the lowest percentage of empty seeds relative to all seeds. Furthermore, this accession and 30/46/27 exhibited the most minimal variation in the values of this parameter. Nevertheless, the lowest disparity between the minimum and maximum percentage of empty seeds was still observed in the control plants. However, in the M2 generation, a significantly lower yield of mature seeds was obtained than in the M1 generation (Figure 3D). The yield from control plants was also minimal and was the lowest among the plants under study. The highest number of seeds was obtained from one plant from accession 30/9/1, which produced 1390 seeds, and from accession 40/27/6 (1042 seeds). The remaining plants exhibited an average yield ranging from 310 to 453 seeds. Furthermore, seed mass was found to be more uniform across these plants than in the M1 generation (see Figure 3E). The greatest variation between accessions was observed in terms of seed weight. The maximum recorded weight was observed in the control plants (0.0408 g), while the minimum was recorded in accession 40.27/6 (0.0325 g). The mean weight for accession 40.27/6 was 0.0271 g (Figure 3F). In summary, the most optimal yield in terms of both the number and weight of seeds was observed in accession 40/27/6 in the M2 generation. However, it was noted that the weight of a single seed was the least substantial.

### 2.3. DNA Sequence Variation

**Figure 4:**
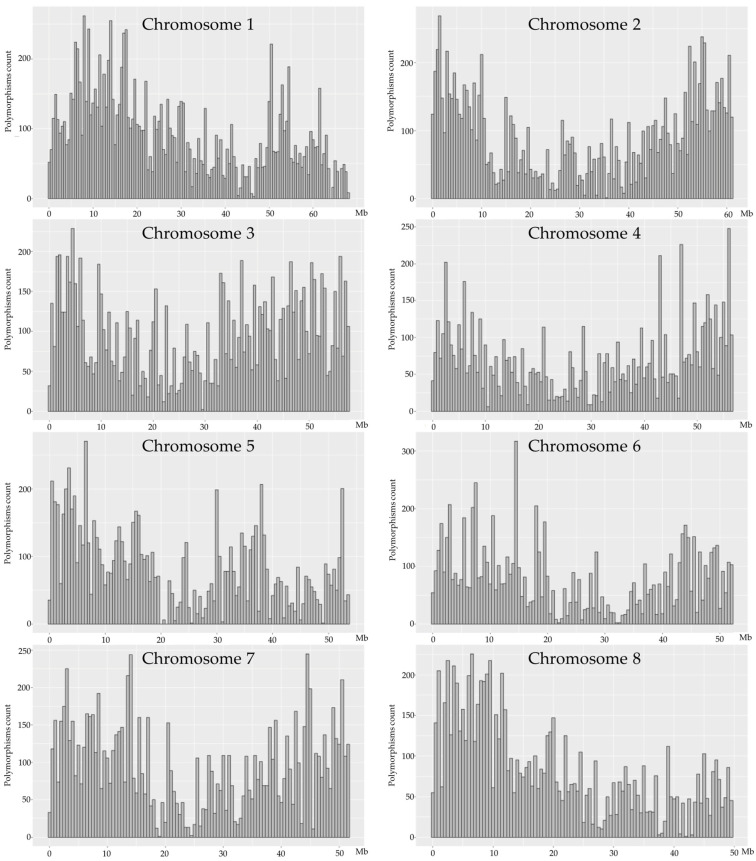
The chromosomal distribution of nGBS sequence polymorphisms detected in one common buckwheat plant from M0 group among five analyzed plants. The window size employed for this analysis was 500,000 base pairs.

**Figure 5:**
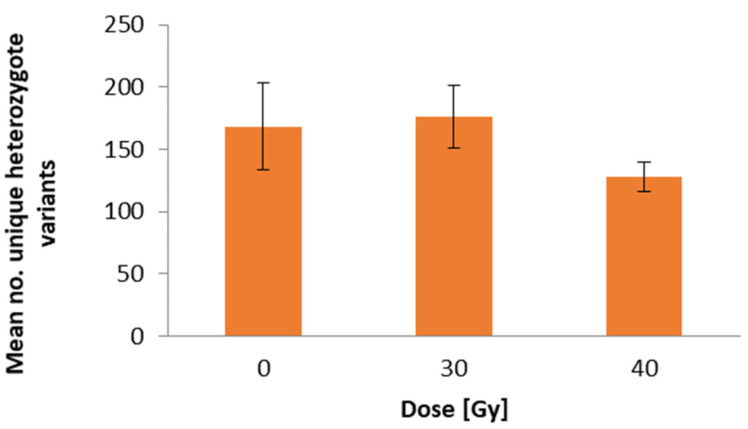
The subsequent figure details the number of unique heterozygote variants identified for the control plants (0 Gy) and the plants of M0 obtained from irradiated seed (30 and 40 Gy). The data are presented as the means (*n* = 5) ± SE.

For all the samples analyzed in this study (from five plants of each group), a total of 54.27 million raw reads were generated, which corresponded to 7.12 gb of sequence data. The raw reads exhibited an average length of 143 nucleotides (nt). The Phred quality score (Q) ranged from 35 to 73, with an average of 67. The parameters Q20 and Q30 reached 95.8% and 91%, respectively. The GC content was found to be 40%. Following the filtration process implemented by the NGS provider, a total of 49.72 million reads were retained, amounting to 91.6% of the initial sequence. This equates to an average of 3.31 million filtered reads per sample. A comparison of the sequence data with the reference genome enabled the identification of approximately 200,000 sequence polymorphisms, which exhibited a valley-shaped distribution along chromosomes (Figure 4). The character of sequence changes in M0 vs. control plants was taken into account, and this pool of polymorphisms was filtered for unique (present in only one analyzed plant) heterozygote variants. Of these variants, 842 were found among the control plants, 880 among the 30 Gy plants, and 639 among the 40 Gy plants (Figure 5).

### 2.4. Amino Acid Content

In the seeds, the following amino acids were determined: asparagine, threonine, serine, glutamine, proline, glycine, alanine, valine, isoleucine, leucine, tyrosine, phenylalanine, histidine, lysine, arginine, cysteine. The results in Table 2 demonstrate that both radiation treatments reduced the total content of amino acids in the seeds of the M2 generation compared to that of the control. However, changes in the percentage content of individual amino acids in the total amino acid pool of each accession were not reflected in a decrease in their individual content compared to that of the control. The seeds of the control plants exhibited the highest percentage amounts of asparagine, serine, proline, phenylalanine, and arginine, while the mutants demonstrated higher percentage amounts of other amino acids. For instance, the percentage amount of glycine increased in the seeds of all selected accessions, irrespective of the radiation dose, when compared to the control (Table 2). However, the absolute content of this amino acid was significantly lower than in the control seeds (Figure 4). The seeds of the 30/46/27 accession exhibited the highest percentage amounts of threonine, glycine, alanine, valine, isoleucine, leucine, tyrosine and cysteine (Table 2). Conversely, the radiation effect on amino acid percentage content in the total amino acid pool of M2 generation exhibited a decline, as evidenced by the decrease in asparagine, glutamine, proline, valine, leucine, phenylalanine, cysteine, methionine, arginine, and serine (Table 2). Conversely, an increase in the percentage content in the seeds of M2 generation independent of radiation dose was observed in the case of tyrosine, glycine, alanine, histidine, methionine and lysine.

**Figure 6:**
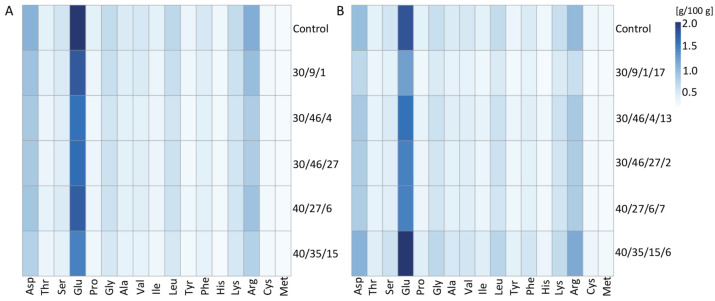
Heat maps demonstrating individual amino-acid content in the seeds [g g^–1^ DW] of chosen mutants of M2 (**A**) and M3 (**B**) generations of common buckwheat. Asp—asparagine; Thr—threonine; Ser—serine; Glu—glutamine; Pro—proline; Gly—glycine, Ala—alanine; Val—valine; Ile—isoleucine; Leu—leucine; Tyr—tyrosine; Phe—phenylalanine; His—histidine; Lys—lysine; Arg—arginine; Cys—cysteine; Met—methionine. The numerals 30 and 40 refer to the radiation dose, with the subsequent number denoting the individual plant from which seeds were obtained.

The weakest impact of both radiation doses on absolute value was detected in the case of cysteine and methionine content (Figure 6A and Supplementary Figure S1). A more pronounced effect of radiation in this generation on the remaining amino acids was observed, for example, on glutamine.

In the M3 generation, the seeds of accession 30/9/1/17 exhibited the highest percentage of threonine, alanine, valine, isoleucine, leucine, tyrosine and leucine compared to the control and other plants (Table 3). Concurrently, this accession demonstrated the lowest content of total amino acids. Conversely, in the M2 generation, the seeds of the 30/9/1 accession exhibited the highest content of most amino acids when compared to the other plants. However, in the M3 generation, the content of amino acids in the seeds of this accession was the lowest (Figure 6B and Supplementary Figure S1).

In the case of accession 40/35/15/6, the total content of amino acids was the highest, although this was not reflected in the increased percentage of most amino acids. As demonstrated in both Figure 6B and Supplementary Figure S1, the absolute content of all amino acids except isoleucine in the seeds of this accession was the highest when compared to that of the control and other accessions. In the seeds of the control plants, the percentage content of glycine, tyrosine, lysine, cysteine and methionine was the lowest when compared to the mutant plants (although in the case of glycine and lysine, the share of these amino acids was the same in the seeds of the 40/35/15/6 accession). It is noteworthy that the total content of amino acids in the seeds of the M2 generation of this accession was the lowest, and in M3 it was the highest compared to the other accessions and the control (Table 2 and Table 3).

## 3. Discussion

In the present study, the impact of two distinct radiation doses on the germination capacity of seeds, leaf area, and seed yield was examined. The application of 40 Gy of radiation resulted in a significant reduction in the number of seeds that germinated when compared to both the control seeds and those exposed to 30 Gy. A dose of 30 Gy resulted in an initial stimulation of leaf area during the early stages of plant development, while no significant differences were observed in the later growth period. A comparable effect of radiation on the development of common buckwheat leaves was previously observed by Mathur [23], who, in contrast, employed considerably higher doses ranging from 5 kR to 40 kR (50 Gy to 400 Gy). Mathur [26] demonstrated that gamma-ray irradiation of buckwheat seeds resulted in a decline in their germination capacity as the radiation dose increased. This relationship was sustained in the subsequent two generations of buckwheat. In the present studies, both radiation doses were found to stimulate plant growth and increase the number of first-class branches, which may account for the higher seed yield. The enhancement in plant height and the number of branches was also observed to persist in the M2 and M3 generations. Mathur [26] obtained similar results for F. esculentum, but not for F. tataricum. Notably, the author observed the greatest number of lateral shoots as an effect of radiation in the third generation. In a separate study, Jia and Li [27] irradiated the F. dibotrys rhizomes with gamma rays of intensity ranging from 5 to 20 Gy. A significantly reduced plant stature with a reduced number of branches was observed as a consequence of a dose as low as 5 Gy. This outcome suggests that the low-dose radiation may have exerted its effect on the rhizomes themselves rather than on the seeds, which are known to have a hard coat that acts as a protective layer for the embryo.

In a subsequent study, Tang et al. [28] investigated the impact of gamma radiation in the range of 100–800 Gy on the seed germination, seedling height and development, and root system of Tartary buckwheat. The authors demonstrated that the mutation frequency was elevated following seed irradiation with doses of 700–800 Gy, while doses of 100–200 Gy induced no mutational effect.

Mathur [23] asserts that irradiating seeds frequently results in the cultivation of dwarf forms. The author reported that the dwarf buckwheat forms exhibited limited growth but better leaf and root development. Furthermore, mutants displayed short internodes, increased floral production, and elevated seed yield. In the present study, only a few accessions were found to be significantly shorter, but they exhibited poor flowering and minimal yield. Consequently, these plants were eliminated from the study. Notably, irrespective of the radiation dosage, all plants flowered concurrently. Mathur [23] observed a different effect, finding that radiation doses of up to 150 Gy accelerated the flowering date in plants. Conversely, a decline in all biometric parameters was observed following the treatment with 400 Gy dose.

In the present study, radiation at doses of 30 and 40 Gy increased the number of empty and ripe seeds, as well as their weight, in the M0 generation. However, the increase in single seed weight was only observed in a limited number of plants. In general, 40 Gy radiation increased seed yield. In the subsequent M1 generation, a comparatively minor disparity in yield parameters was observed between control and mutant plants. In the M2 generation, the percentage of empty seeds was comparable for plants from both treatments, yet the yield of mature seeds was higher for plants from the 40 Gy group relative to the yield of control plants and accessions from the 30 Gy group. The weight of a single seed from the control plants was considerably higher, confirming our previous results showing that in common buckwheat the higher the number of seeds, the lower the weight of a single seed [20]. This correlation was observed to persist across all the generations studied, suggesting that in common buckwheat, this relationship is unbreakable. As posited by Oh et al. [29], the correlation between seed size and threshing efficiency is not the sole determining factor in buckwheat yield. It was demonstrated that larger seeds generally contribute to heavier yields, leading to higher overall production. Mathur [23] obtained the highest yield per plant (number of seeds and seed weight) after the application of 100 and 150 Gy for both common buckwheat and Tartary buckwheat. This effect was sustained at a comparable level across three generations. A comparison of these results with those obtained in our study suggests that a lower radiation dose may be effective in increasing yield, however, it is still unstable. In addition to genetic factors, yield is found to be strongly influenced by temperature and water availability during the vegetation period. In the M2 generation, a significantly smaller yield of mature seeds was obtained than in the M1 generation. The yield from control plants was also small and was the lowest among the plants under study. The underlying cause of this phenomenon could be attributed to the prolonged periods of elevated temperatures experienced during the current growing season.

Ahmad et al. [30] induced mutagenesis in Tartary buckwheat seeds using gamma rays at doses ranging from 50 to 250 Gy, as well as ethyl methanesulfonate (EMS) at concentrations of 0.1%, 0.2%, 0.3%, 0.4%, and 0.5% *w*/*v*. Their analysis of variance revealed that key phenotypic traits—including plant height, number of inflorescences, days to flowering, and seed yield—varied significantly between the mutant populations and the parent population. The results demonstrated a strong potential for selection to improve yield traits in Tartary buckwheat, as indicated by the significant genotypic coefficient of variation (GCV), high heritability (h^2^), and substantial genetic advance (GA) induced by the mutagenic treatments in the selected populations. In our experiment, the nGBS (normalized genotyping-by-sequencing) analysis was conducted to ascertain whether irradiation led to the formation of heterozygote variants in the common buckwheat plants obtained from gamma-exposed seeds. Although sectorial (chimerism), such variants lead to the formation of mutant homozygotes in further generations, allowing for a phenotypic expression of the modified genes [31]. In our work, we assumed that such treatment-induced heterozygotes should be rare and therefore, we selected the fraction of those which were present in only one plant from the analyzed set. However, it appears that in our data, such heterozygotes were masked by the extensive background heterozygosity of buckwheat, as reflected in the total number of sequence polymorphisms and their chromosomal distribution. The latter displayed minima, similarly to what has been observed in other plant species, e.g., by Baguma et al. [32]. Such minima likely correspond to the locations of centromeres, as they are enriched in repetitive sequences that are largely eliminated during GBS library construction. Likewise, the observed patterns of polymorphism distribution correlate with the fact that Fagopyrum chromosomes are mostly metacentric [33]. It should be emphasized that in the case of common buckwheat, which is not only heterozygous but also characterized by heterostyly, the effect of the mutation is definitely more difficult to determine than in the case of self-pollinating Tartary buckwheat. It is not surprising that many studies of mutagenic variability have been carried out on this Fagopyrum species.

In the present study, the percentage share in the total pool, as well as the absolute content of amino acids in the seeds of individual accessions, was found to differ between the M2 generation and the M3 generation. Of particular interest are the amino acids that distinguish common buckwheat as a notable source, namely lysine and arginine. While the seeds of the M2 generation of the 40/35/15 genotype exhibited a reduced content of individual amino acids compared to the control, in the M3 generation the content of most amino acids in the seeds of its M3 generation (40/35/15/6) was significantly higher than in the seeds of the other accessions and control plants. The elevated amino acid content encompassed not only arginine and lysine, but also cysteine, phenylalanine, tyrosine, and threonine. Most proteins in common buckwheat are globulins and albumins, containing a wide range of different amino acids [34]. According to these authors, common buckwheat seeds contain high levels of lysine 6.1%, arginine 9.7%, aspartic acid 11.3% and low content of proline (3.9%) and glutamic acid (18%). Our studies confirmed that, in the M2 and M3 generations of common buckwheat, proline was about 3.7%. In the M3 generation, we obtained a higher content of lysine 6.62%, arginine 10.47% and glutamine 19.48%. These results confirm that treatment of seeds with gamma rays, even at a low dose compared to the higher doses used by other authors, stimulates an increased content of essential amino acids.

In a study by Mohamed et al. [35], the effects of 2 kGy radiation on the total protein and amino acid composition of raw and processed flour from two pearl millet cultivars during storage were investigated. The study revealed that the majority of amino acids exhibited stability to this treatment, with the exceptions of leucine, glutamic acid and phenylalanine. Notably, the amino acid content of one cultivar exhibited an increase following irradiation, irrespective of storage duration and processing method.

As demonstrated in the study by Hooshmand and Kloopfenstein [36], the irradiation of ground grains at doses ranging from 5 to 20 kGy resulted in a decline of lysine, albeit a minor one, in corn, wheat, and soybeans. Concurrently, methionine levels diminished in wheat and corn grains. In some cases, a decrease in phenylalanine was observed in corn and wheat, and a decrease in histidine was observed in wheat. However, the study noted that other essential amino acids remained largely unaffected by the irradiation process. These findings imply a notable stability of the amino acid composition in the ground grain, even following exposure to a substantial dose of radiation. In the present study, the alterations in the amino acid composition of buckwheat seeds were observed to be a consequence of radiation following a cultivation period of three years, with successive generations being subjected to the same conditions. However, it is challenging to unequivocally ascertain whether these alterations were initiated by genetic modifications or the impact of environmental factors during the growth period. The findings of Oh et al. [29] merit attention, as they demonstrated a multifaceted inheritance pattern of traits such as seed size and seed coat color, contingent on the pin and thrum flower type of common buckwheat, and the interplay between genetic background and environmental conditions. In contrast, Tang et al. [28] reported a significant increase in the level of flavonoids in the seeds of Tartary buckwheat following the irradiation of seeds with doses ranging from 200 to 600 Gy. It is important to note, however, that Tartary buckwheat is a self-pollinating plant and does not exhibit heterostyly, which more complicates the fertilization process in common buckwheat.

## 4. Materials and Methods

### 4.1. Plant Material

The experiment was conducted on Polish buckwheat cultivar ‘Korona’ The seeds were delivered from Małopolska Plant Breeding Centre in Polanowice (Slomniki, Poland).

### 4.2. Study Design

The experiment was conducted over a period of three years, from 2021 to 2024. In 2021, the seeds were exposed to 30 or 40 Gy of gamma rays, subsequently sown into pots, and cultivated within an open foil tunnel. Mutants (M0) were then cross-pollinated under isolation conditions within groups, depending on the mutation method, to obtain M1 (2022), M2 (2023), and M3 (2024) generations. In subsequent years, plants were selected within M0, M1, M2 and M3 based on seed yield. The following parameters were analyzed each year: morphological characteristics (biometric parameters: height of stems, number of first-class branches) and seed yield (number of ripe and empty seeds, mass of seeds produced by single plant, mass of one seed, mass of one thousand seeds). In the M0 group, the percentage of germinating seeds after gamma irradiation, the area of well-developed leaves of the third node, and the fresh and dry weight of the above-ground parts of the plants were analyzed. Normalized genotyping-by-sequencing (nGBS) was performed on selected mutants of M0, which were characterized by the highest seed production.

Subsequently, the amino acid composition of seeds from selected plants in the M2 and M3 generations was determined. Seeds from M1 and M2 plants were treated as M2 and M3 generation, respectively. Seeds from the M3 generation were only analyzed in terms of amino acid content. The number of accessions analyzed in terms of biometric parameters and amino acid content are presented in Table 4 and Table 5, respectively.

### 4.3. Seed Irradiation

The seeds were immersed in water for a period of 24 h, after which they were exposed to gamma rays at a dose of 30 or 40 Gy (500 seeds per dose). This procedure was carried out at the Institute of Oncology in Kraków, Poland, by specialists in the field of radiology. The technical data for this experiment is as follows: The linear accelerator used was a Varian medical linear accelerator of the Silhouette type (Varian Medical Systems, Palo Alto, CA, USA); 6 MV high-energy photons were used, representing the most popular energy type in the world. Irradiation of the plant material was carried out in PMMA blocks, a method that allows for accurate delivery of the dose and ensures uniformity across the entire area. The irradiation field is adapted to the size of the dishes (25 cm × 25 cm), and the distance from the radiation source is 100 cm.

### 4.4. Seed Germination and Plant Cultivation

The seeds, which had undergone the process of irradiation and were used as a control (200 seeds of each group), were sown into pots (with a quantity of four seeds per pot). The percentage of seeds that germinated was calculated. The pots were filled with a mixture of commercial soil substrate (pH 6.0; Eko Ziem, Jurków, Poland) with perlite in a ratio of 1:1 (*v*:*v*). The plants were cultivated in an open foil tunnel at the University of Agriculture in Kraków (Poland), which is located at 50°04′10″ N and 19°50′44″ E. The vegetation period for the plants lasted from 20 May to 10 September each year of the experiment. The plants were fertilized with Hoagland’s nutrient solution [37] once a week. Due to the necessity of cross-pollination between plants with pin and thrum flowers, plants within each radiation dose treatment group were cultivated under separate isolators. As common buckwheat is insect-pollinated, fly larvae (*Calliphora vicina*) were placed under the isolators, from which adult pollinating insects subsequently hatched. Accessions of M0, which exhibited the highest number and weight of seeds obtained from one plant, the lowest percentage of empty seeds and a relatively high weight of single seeds, were selected for further studies. Seeds from these accessions (progeny named M1) were then collected and sown into pots (40 seeds of each accession, 4 seeds per pot) in the following year of study (Table 1). The mean dose was 30 and 40 seeds, respectively, and the number of individual plants from which seeds were obtained. Each plant received a unique identifier. The progeny of each accession was cultivated separately under isolators. The same selection and breeding procedure was used in the M2 generation.

### 4.5. Biometric Analyses of M0 Generation

Well-developed leaves of the third node above the ground were cut and their surface was measured using CI—202 Portable Laser leaf Area Meter (CID Bio-Science, Camas, WA, USA). The analysis was performed on 30 leaves for each plant group. At the end of the growing season, when the plants had not yet begun to desiccate, the following biometric analyses were conducted: stem height, the number of branches, and the fresh and dry weight of stems (FW and DW), respectively. Additionally, the number of empty and ripe seeds per plant, as well as the weight of a single seed, were recorded. Depending on the number of germinated seeds and plant survival of each group, these analyses were carried out on a different number of plants, and 20 control plants. All plants were numbered, and the mutants obtained from the irradiated seeds were designated the M0 generation (e.g., 30/1 or 40/4). The selection of the highest-yielding plants (i.e., those producing the most mature seeds, with the highest weight, and at the same time showing the lowest percentage of empty seeds) was conducted for further study; that is to say, their seeds, which were the M1 generation, were harvested and sown the following year.

### 4.6. Biometric Analyses of M1 and M2 Plants

The M1 and M2 generations were analyzed in the same manner as the M0 generation, with the exception of seed germination, leaf area, FW and DW of stems. Seeds were collected from individual plants within each group. Following the evaluation of the yield parameters of the plants, the best yielding ones were selected, i.e., those showing the highest number of seeds, their weight, and the lowest percentage of empty seeds. The seeds from these plants were then sown the following year in separate groups (i.e., progeny obtained from a single plant) under isolators to facilitate cross-breeding. Subsequent to this, the aforementioned parameters were evaluated in order to ascertain whether the given traits were maintained in the subsequent generation and to determine the dispersion of the evaluated parameters.

### 4.7. NGS-Based Genotyping

Total cellular DNA was isolated from leaf fragments, which were pulverized in liquid nitrogen. Approximately 1 g of the resulting powder was suspended in 7 cm^3^ of the extraction buffer (4% CTAB, 1.4 M NaCl, 20 mM EDTA, 1% PVP, 0.1% 2-mercaptoethanol, 100 mM Tris HCl pH 8.0). The samples were then supplemented with 6 cm^3^ of a chloroform/isoamyl alcohol mixture (24: 1, *v*/*v*) and manually shaken for 5 min. This was followed by centrifugation at 17,100× *g* for 10 min. Subsequently, 5 cm^3^ of the aqueous phase was collected into a new tube and supplemented with the same volume of room-temperature isopropanol. Following brief mixing, the samples were subjected to centrifugation as previously described. The upper layer was then discarded, and the lower layer was transferred into a 2 cm^3^ tube and rinsed with 1.5 cm^3^ of ethanol. Subsequently, the samples were subjected to 5 min. centrifugation at 18,200× *g*, after which the supernatants were discarded. The DNA pellets were then dried in a vacuum desiccator (10–15 min.) and dissolved in 300 μL of buffer TE. A 100-μL portion of the resulting DNA sample was then supplemented with 10 μL of RNase A (10 µg/cm^3^, cat. no. R4875, Merck, Darmstadt, Germany) and incubated for 1 h at 37 °C. Subsequent purification of the DNA was accomplished through the utilization of solutions from Wizard SV Gel (Promega, Madison, WI, USA) and PCR Clean-Up System (Promega, Madison, WI, USA), in conjunction with Mini Spin Columns Ⅲ (Dongsheng Biotech, Guangzhou, China). Elution was performed with 50 μL of water. Subsequent to quality control in a spectrophotometer NanoDrop 2000c (Thermo Fisher Scientific, Wilmington, DE, USA) and by standard agarose electrophoresis, the samples were dispatched to LGC Genomics GmbH (Berlin, Germany) for the purpose of normalized genotyping-by-sequencing (nGBS) [38]. For library construction, the MslI restriction enzyme was utilized. PE150 Illumina sequencing was performed with the NextSeq 500/550 v2 Kit (Illumina, San Diego, CA, USA). Subsequent to this, the service provider subjected the sequence data to demultiplexing, adapter clipping and restriction site filtering. Following the acquisition of the data from LGC Genomics GmbH, the sequence reads were subjected to mapping using BWA [39] to the reference genome (GenBank: GCA_002319775.1), resulting in the creation of SAM files. These were then converted to BAM files using SAMtools (v1.9) [40]. The software was also used for the sorting and indexing of the BAM files, which were then analyzed with Platypus [41]. The resulting VCF file was then subjected to a process of filtration, whereby low-quality polymorphisms were eliminated with the use of VCFtools (v0.1.16) [42]. Following this filtration process, a custom Python (v.3.8.1) script (designed for personal use by W. Wesołowski) was utilized to extract heterozygote variants that were unique to a single sample (plant). The analyses were conducted on five plants for each plant group (control, 30 and 40 Gy).

### 4.8. Amino Acids Assay in the Seeds

Amino acid analysis was conducted in accordance with the methodology established by Moore and Stein [43,44,45]. The milled samples were hydrolyzed in the liquid phase (6 M HCl at 110 °C for 24 h under an inert gas atmosphere). The hydrolysates were then lyophilized, following which they were dissolved in sodium citrate buffer (pH 2.2). The solution was filtered through a 0.45 μm filter. The amino acid composition was then analyzed by ion-exchange chromatography with post-column ninhydrin derivatization reaction and spectrophotometric detection (570 and 440 nm) in accordance with the manufacturer’s standard procedure (Ingos, Prague, Czech Republic). Sulfur-containing amino acids were analyzed as oxidation products obtained by performic acid oxidation followed by the above hydrolysis procedure. A standard amino acid solution (Merck, Darmstadt, Germany) was used as a calibrator, and measurement data were evaluated using Chromulan software v.0.90 (Pikron, Prague, Czech Republic). During the process of acid hydrolysis, asparagine is converted to aspartic acid and glutamine to glutamic acid. Therefore, the analysis result for Asp = Asp + Asn, and the result for Glu = Glu + Gln. It is important to note that the amino acid composition determined in this study does not include tryptophan, which is destroyed during the hydrolysis process.

### 4.9. Statistical Analyses

The analysis of leaf area and the number of unique heterozygote variants was conducted using a one-way analysis of variance (ANOVA) and a post hoc Tukey test (at *p* < 0.05). The statistical software used was STATISTICA 13.0 (Stat-Soft, Inc., Tulsa, OK, USA). The data were represented as means ± standard error (SE). The significance of differences between amino acid contents was marked by the least significant difference (LSD) at *p* < 0.05. The results of FW and DW, the number of mature and immature seeds, the weight of seeds and also single seed weight measurements were presented in Tukey’s box plots. To compare outcomes measured in agronomic traits between groups of plants, the Mann–Whitney U test (*p* > 0.05) was used. The statistical analysis and data visualization were performed using R 4.4.2 [46], caret (v6.0–94) [47], ggplot2 (v3.5.1) [48] and rstatix (v0.7.2) [49].

## 5. Conclusions

The irradiation of common buckwheat seeds with doses of 30 and 40 Gy does not result in the cultivation of dwarf plants; however, it has been observed to enhance both the height of the plants and the number of first-class branches. Conversely, the enhanced seed yield was not consistently observed in all the plants examined and was frequently not replicated in subsequent generations. However, a smaller dispersion of the evaluated traits was obtained following pollination within the progeny of selected, best-yielding plants, in comparison to the initial generation (M0 group; i.e., plants grown from irradiated seeds). For this reason, we selected seeds only from the best-yielding single plants for further studies in subsequent generations. The analysis of the obtained results suggests that the effect of mutations in heterogeneous common buckwheat should be assessed at the earliest in the M3 generation. A strong interaction between genotype and environmental conditions persists, as does a correlation between the number of ripe and empty seeds obtained from a single plant. These observations suggest these traits are strongly conjugated and will be challenging to modify by genetic manipulation. The treatment of seeds with 40 Gy significantly reduces the percentage of germinating seeds; however, it is more effective in increasing seed yield and amino acid content. To effectively monitor mutational changes in the M0 generation, whole genome re-sequencing is recommended as GBS only targets a relatively small part of the genome. The heterostyly and self-incompatibility of common buckwheat make detection of mutation changes much more difficult compared to Tartary buckwheat, which is self-pollinating. Consequently, it would be advisable to employ higher doses of radiation to achieve greater genetic variability. However, higher doses of radiation greatly reduce the percentage of germinated seeds, so large quantities of seeds must be irradiated and the area under cultivation increased.

We would like to point out that analyses of the embryological development in the flowers of three generations of mutant buckwheat and the content of phenolic compounds in the seeds are currently underway.

## Figures and Tables

**Table 1:** A comparison of the leaf area of plants that were treated with 30 Gy and 40 Gy of seed treatment. The measurement was conducted five weeks after the initial sowing of the seeds. The means (*n* = 30) ± SE were marked with the same letter did not differ significantly (Tukey test at *p* < 0.05).

Treatment	Leaf Area [cm^2^]
Control	65.9 ± 3.2 ^b^
30 Gy	71.8 ± 4.7 ^ab^
40 Gy	55.6 ± 2.7 ^c^

**Table 2:** The percentage [%] content of individual and total amount [g g^−1^DW] of amino acids is presented as the mean (*n* = 6) in the seeds of selected accessions of M2 common buckwheat generation obtained after seed gamma radiation with 30 or 40 Gy. The LSD (least significant difference) was used to determine statistical significance.

Amino Acid	Control	30/9/1	30/46/4	30/46/27	40/27/6	40/35/15	LSD
Asp	9.98	9.92	9.91	9.86	9.90	9.78	0.01
Thr	3.96	4.04	4.00	4.05	3.96	3.99	0.03
Ser	5.28	5.24	5.19	5.14	5.24	5.19	0.02
Glu	19.17	18.68	18.56	18.49	19.20	18.66	0.04
Pro	3.82	3.73	3.64	3.76	3.76	3.68	0.03
Gly	6.27	6.52	6.62	6.66	6.41	6.56	0.04
Ala	4.57	4.66	4.74	4.78	4.56	4.64	0.05
Val	5.02	4.95	4.98	5.06	4.89	4.82	0.01
Ile	3.75	3.70	3.74	3.80	3.65	3.62	0.02
Leu	6.63	6.59	6.61	6.68	6.50	6.55	0.01
Tyr	3.03	3.10	3.17	3.19	3.12	3.17	0.01
Phe	4.77	4.67	4.66	4.71	4.63	4.58	0.02
His	2.75	2.89	2.87	2.87	2.76	2.82	0.01
Lys	6.11	6.34	6.38	6.32	6.19	6.35	0.02
Arg	10.26	10.08	9.44	9.23	10.19	9.83	0.03
Cys	2.53	2.70	2.86	2.92	2.71	3.08	0.04
Met	2.11	2.19	2.64	2.48	2.34	2.69	0.04
Total amount	10.46	9.25	8.37	8.53	8.86	8.53	0.03

Asp—asparagine; Thr—threonine; Ser—serine; Glu—glutamine; Pro—proline; Gly—glycine, Ala—alanine; Val—valine; Ile—isoleucine; Leu—leucine; Tyr—tyrosine; Phe—phenylalanine; His—histidine; Lys—lysine; Arg—arginine; Cys—cysteine; Met—methionine. The numerals 30 and 40 refer to the radiation dose, with the subsequent number denoting the individual plant from which seeds were obtained.

**Table 3:** Percentage [%] content of individual and total amount of amino acids [g g^−1^DW] presented as means (*n* = 6) ± SE in the seeds of selected plants of M3 common buckwheat generation obtained after seed gamma radiation with 30 or 40 Gy. LSD (least significant difference) was used to determine statistical significance.

Amino Acid	Control	30/9/1/17	30/46/4/13	30/46/27/2	40/27/6/7	40/35/15/6	LSD
Asp	9.98	9.89	9.84	9.75	9.80	9.77	0.01
Thr	3.96	4.26	3.97	3.99	4.04	3.85	0.02
Ser	5.28	5.18	5.03	5.10	5.15	5.16	0.02
Glu	19.17	18.16	18.92	18.39	18.70	19.48	0.03
Pro	3.82	3.71	3.77	3.69	3.75	3.71	0.01
Gly	6.27	6.60	6.63	6.56	6.64	6.28	0.01
Ala	4.57	4.97	4.61	4.70	4.68	4.39	0.02
Val	5.02	5.09	4.92	4.96	4.93	4.90	0.02
Ile	3.75	3.82	3.71	3.76	3.72	3.66	0.01
Leu	6.63	6.84	6.60	6.66	6.68	6.56	0.03
Tyr	3.03	3.28	3.24	3.15	3.22	3.27	0.01
Phe	4.77	4.64	4.64	4.67	4.66	4.93	0.01
His	2.75	2.85	2.93	2.77	2.85	2.65	0.02
Lys	6.11	6.62	6.50	6.33	6.33	6.11	0.02
Arg	10.26	9.15	9.58	9.51	9.83	10.47	0.03
Cys	2.53	2.64	2.67	2.65	2.65	2.65	0.01
Met	2.11	2.30	2.43	2.21	2.33	2.14	0.01
Total amount	10.46	7.35	9.26	8.53	8.72	11.67	0.03

Asp—asparagine; Thr—threonine; Ser—serine; Glu—glutamine; Pro—proline; Gly—glycine, Ala—alanine; Val—valine; Ile—isoleucine; Leu—leucine; Tyr—tyrosine; Phe—phenylalanine; His—histidine; Lys—lysine; Arg—arginine; Cys—cysteine; Met—methionine. The numerals 30 and 40 refer to the radiation dose, with the subsequent number denoting the individual plant from which seeds were obtained.

**Table 4:** The number of accessions used in the study of biometric and yield parameters of each generation of mutant plants. The term ‘30 and 40 Gy’ refers to the radiation dose, while the number following the slash denotes the number of individual plants from which seeds were obtained.

Year	Generation	Treatment/No of Accessions		
2021	M0	Control	30 Gy	40 Gy
2022	M1	Control	30/46 30/131 30/9 30/33	40/11 40/18 40/20 40/23 40/27 40/35
2023	M2	Control	30/9/1 30/46/4 30/46/27	40/27/6 40/35/15

**Table 5:** The number of accessions used in the analysis of amino acid content in the seeds. M2 denotes seeds collected from plants of the M1 generation, while M3 refers to seeds obtained from plants of the M2 generation. The term ‘30 and 40 Gy’ refers to the radiation dose, while the number following the slash denotes the number of individual plants from which seeds were obtained.

Year	Generation	Treatment/No of Accessions		
		Control	30 Gy	40 Gy
2023	M2		30/9/1 30/46/4 30/40/27	40/27/6 40/27/1 40/35/15
2024	M3	Control	30/9/1/17 30/46/4/13 30/46/27/2	40/27//6/7 40/35/15/6

## Data Availability

The original data presented in this study are included in the article. Further inquiries can be directed to the corresponding author.

## Supplementary Materials

The following supporting information can be downloaded at https://www.mdpi.com/article/10.3390/ijms26104587/s1.

## Abbreviations

Asp	asparagine
Thr	threonine
Ser	serine
Glu	glutamine
Pro	proline
Gly	glycine
Ala	alanine
Val	valine
Ile	isoleucine
Leu	leucine
Tyr	tyrosine
Phe	phenylalanine
His	histidine
Lys	lysine
Arg	arginine
Cys	cysteine
Met	methionine
nGBS	normalized genotyping-by-sequencing
Q	Phred quality score measure of the quality of the nucleotide identification generated by automated DNA sequencing

## References

[1] Gargano D., Appanna R., Santonicola A., De Bartolomeis F., Stellato C., Cianferoni A., Casolaro V., Iovino P. (2021). Food allergy and intolerance: A narrative review on nutritional concerns. Nutrients.

[2] Khairuddin M.A.N., Lasekan O. (2021). Gluten-free cereal products and beverages: A review of their health benefits in the last five years. Foods.

[3] Christa K., Soral-Śmietana M. (2008). Buckwheat grains and buckwheat products—Nutritional and prophylactic value of their components—A review. Czech J. Food Sci..

[4] Aubert L., Konrádová D., Kebbas S., Barris S., Quinet M. (2020). Comparison of high temperature resistance in two buckwheat species Fagopyrum esculentum and *Fagopyrum tataricum*. J. Plant Physiol..

[5] Domingos I., Bilsborrow P.E. (2022). The effect of variety and sowing date on the growth, development, yield and quality of common buckwheat (*Fagopyrum esculentum* Moench). Eur. J. Agron..

[6] Podolska G., Gujska E., Klepacka J., Aleksandrowicz E. (2021). Bioactive compounds in different buckwheat species. Plants.

[7] Katar D., Olgun M., Turan M. (2016). Analysis of morphological and biochemical characteristics of buckwheat (*Fagopyrum esculentum* Moench) in comparison with cereals. CyTA-J. Food.

[8] Krkošková B., Mrazova Z. (2005). Prophylactic components of buckwheat. Food Res. Int..

[9] Vojtíšková P., Kmentová K., Kubáň V., Kráčmar S. (2012). Chemical composition of buckwheat plant (*Fagopyrum esculentum*) and selected buckwheat products. J. Microbiol. Biot. Food Sci..

[10] Kreft M. (2016). Buckwheat phenolic metabolites in health and disease. Nutr. Res. Rev..

[11] Nešovic M., Gašic U., Tosti T., Horvacki N., Šikoparija B., Nedic N., Blagojevic S., Ignjatovic L., Tešic Ž. (2020). Polyphenol profile of buckwheat honey, nectar and pollen: Polyphenolics in buckwheat. R. Soc. Open Sci..

[12] Farooq S., Rehman R.U., Pirzadah T.B., Malik B., Dar F.A., Tahir I., Zhou M., Kreft I., Woo S.-H., Chrungoo N., Wieslander G. (2016). Cultivation, agronomic practices, and growth performance of buckwheat. Molecular Breeding and Nutritional Aspects of Buckwheat.

[13] Farkas Á., Zajácz E., Spring E., Pasture B.E.E., Summer E. (2007). Nectar production for the Hungarian honey industry. Eur. J. Plant Sci. Biotechnol..

[14] Cawoy V., Kinet J.M., Jacquemart A.L. (2008). Morphology of nectaries and biology of nectar production in the distylous species *Fagopyrum esculentum*. Ann. Bot..

[15] Jacquemart A., Cawoy V., Kinet J.M., Ledent J.F., Quinet M. (2012). Is buckwheat (*Fagopyrum esculentum* Moench) still a valuable crop to day?. Eur. J. Plant Sci. Biotechnol..

[16] Słomka A., Michno K., Dubert F., Dziurka M., Kopeć P., Płażek A. (2017). Embryological background of low seed set in distylous common buckwheat (*Fagopyrum esculentum* Moench) with biased morph ratios, and stimulant- induced improvement of it. Crop Pasture Sci..

[17] Morishita T., Mukasa Y., Suzuki T., Shimizu A., Yamaguchi H., Degi K., Aii J., Hase Y., Shikazono N., Tanaka A. (2010). Characteristics and inheritance of the semidwarf mutants of Tartary buckwheat (*Fagopyrum tataricum* Gaertn.) induced by gamma ray and ion beam irradiation. Breed. Res..

[18] Cawoy V., Ledent J.F., Kinet J.M., Jacquemart A.L. (2009). Floral biology of common buckwheat (*Fagopyrum esculentum* Moench). Eur. J. Plant Sci. Biotechnol..

[19] Inoue N., Hagiwara M. (1999). Analysis of the yielding process based on the module concept in common buckwheat. Fagopyrum.

[20] Hornyák M., Słomka A., Sychta K., Dziurka M., Kopeć P., Pastuszak J., Szczerba A., Płażek A. (2020). Reducing flower competition for assimilates by half results in higher yield of *Fagopyrum esculentum*. Int. J. Mol. Sci..

[21] Mittal H.K., Gautam A.S., Panwar K.S., Jungpo B. (2000). Synergistic effects of combined treatments of gamma-irradiation and ethyl methane sulphonate in buckwheat (*Fagopyrum tataricum*). Ind. J. Agric. Res..

[22] Micke A., Donini B., Maluszynski M. (1990). Induced Mutations for Crop Improvement-A Review.

[23] Mathur A. (1990). Mutation studies in buckwheat (*Fagopyrum*) III. Effect of gamma rays on growth parameters and yield attributes. Fagopyrum.

[24] Kurowska M., Labocha-Pawłowska A., Gnizda D., Maluszynski M., Szarejko I. (2012). Molecular analysis of point mutations in a barley genome exposed to MNU and gamma rays. Mutat. Res. Fundam. Mol. Mech. Mutagen..

[25] Gregori M., Kreft I. (2012). Breakable starch granules in a low-amylose buckwheat (*Fagopyrum esculentum* Moench) mutant. J. Food Agric. Environ..

[26] Mathur A. (1989). Mutation studies in buckwheat (*Fagopyrum*) I. Effect of gamma rays on germination. Fagopyrum.

[27] Jia C.F., Li A.L. (2008). Effect of gamma radiation on mutant induction of *Fagopyrum dibotrys* Hara. Photosynthetica.

[28] Tang Y., Bin Z.X., Gang Z., Maluszynski M., Kasha K.J. (2002). Improvement of tartary buckwheat by induced mutations with ^60^Co gamma rays. Mutations, In Vitro and Molecular Techniques for Environmentally Sustainable Crop Improvement.

[29] Oh M.A., Park J.E., Kim J.Y., Kang H.M., Min Oh S.S., Mansoor S., Chung Y.S. (2024). Seed traits inheritance in *Fagopyrum esculentum* Moench. based on image analysis method. Front. Plant Sci..

[30] Ahmad J.J.S., Javid W., Bhat S.A., Tahir I. (2023). Assessment on induced genetic variability and divergence in the mutagenised Tartary buckwheat populations developed using gamma rays and EMS mutagenesis. Ecol. Gen. Genom..

[31] Parry M.A.J., Madgwick P.J., Bayon C., Tearall K., Hernandez-Lopez A., Baudo M., Rakszegi M., Hamada W., Al-Yassin A., Ouabbou H. (2009). Mutation discovery for crop improvement. J. Exp. Bot..

[32] Baguma J.K., Mukasa S.B., Nuwamanya E., Alicai T., Omongo C.A., Ochwo-Ssemakula M., Ozimati A., Esuma W., Kanaabi M., Wembabazi E. (2024). Identification of genomic regions for traits associated with flowering in cassava (*Manihot esculenta* Crantz). Plants.

[33] Wang L., Sheng M. (2022). Chromosomal location of 45S and 5S rDNA in the strains of *Fagopyrum esculentum* Moench and *F. tataricum* (L.) Gaertn. Cytologia.

[34] Wijngaard H.H., Arendt E.K. (2006). Buckwheat. Cereal Chem..

[35] Mohamed E.A., Mohamed Ahmed I.A., Yagoub A.E.A., Babiker E.E. (2010). Effects of radiation process on total protein and amino acids composition of raw and processed pearl millet flour during storage. Int. J. Food Sci. Technol..

[36] Hooshmand H., Klopfenstein C.F. (1995). Effects of gamma irradiation on mycotoxin disappearance and amino acid contents of corn, wheat, and soybeans with different moisture contents. Plant Foods Human Nutr..

[37] Hoagland D.R., Arnon D.I. (1938). The water-culture method for growing plants without soil. Univ. Calif. Agric. Exp. Station Circ..

[38] Arvidsson S., Fartmann B., Winkler S., Zimmermann W. (2016). Efficient High-Throughput SNP Discovery and Genotyping Using Normalised Genotyping-by-Sequencing (nGBS).

[39] Durbin R., Li H. (2009). Fast and accurate short read alignment with Burrows–Wheeler transform. Bioinformatics.

[40] Li H., Handsaker B., Wysoker A., Fennell T., Ruan J., Homer N., Marth G., Abecasis G., Durbin R. (2009). The Sequence Alignment/Map format and SAMtools. Bioinformatics.

[41] Rimmer A., Phan H., Mathieson I., Iqbal Z., Twigg S.R.F., Wilkie A.O.M., McVean G., Lunter G. (2014). Integrating mapping-, assembly- and haplotype-based approaches for calling variants in clinical sequencing applications. Nat. Genet..

[42] Danecek P., Auton A., Abecasis G., Albers C.A., Banks E., DePristo M.A., Handsaker R.E., Lunter G., Marth G.T., Sherry S.T. (2011). The variant call format and VCFtools. Bioinformatics.

[43] Moore S., Stein W.H. (1951). Chromatography of amino acids on sulfonated polystyrene resins. J. Biol. Chem..

[44] Davidson I., Smith B.J. (2003). Hydrolysis of samples for Amino Acid Analysis. Protein Sequencing Protocols.

[45] Smith A.J., Smith B.J. (2003). Post Column Amino Acid Analysis. Protein Sequencing Protocols.

[46] R Core Team (2022). R: A Language and Environment for Statistical Computing.

[47] Kuhn M. (2008). Building predictive models in R using the caret package. J. Statist. Soft..

[48] Wickham H. (2016). ggplot2: Elegant Graphics for Data Analysis.

[49] Kassambara A. rstatix: Pipe-Friendly Framework for Basic Statistical Tests. https://rpkgs.datanovia.com/rstatix.

