# Supplementary material for: Higher Yield of Common Buckwheat (Fagopyrum esculentum Moench) as a Result of Seed Treatment with Gamma Radiation
Source: Int J Mol Sci. 2025 May 10;26(10):4587. doi: 10.3390/ijms26104587 (PMC12110951; doi:10.3390/ijms26104587)
Supplement: Supplementary file 1 [file ijms-26-04587-s001.zip › Figure S1.pdf]

Supplementary Figure S1. Amino acid content in the seeds of selected mutant accessions of common buckwheat of the M2 and M3 generations.

M2 M3

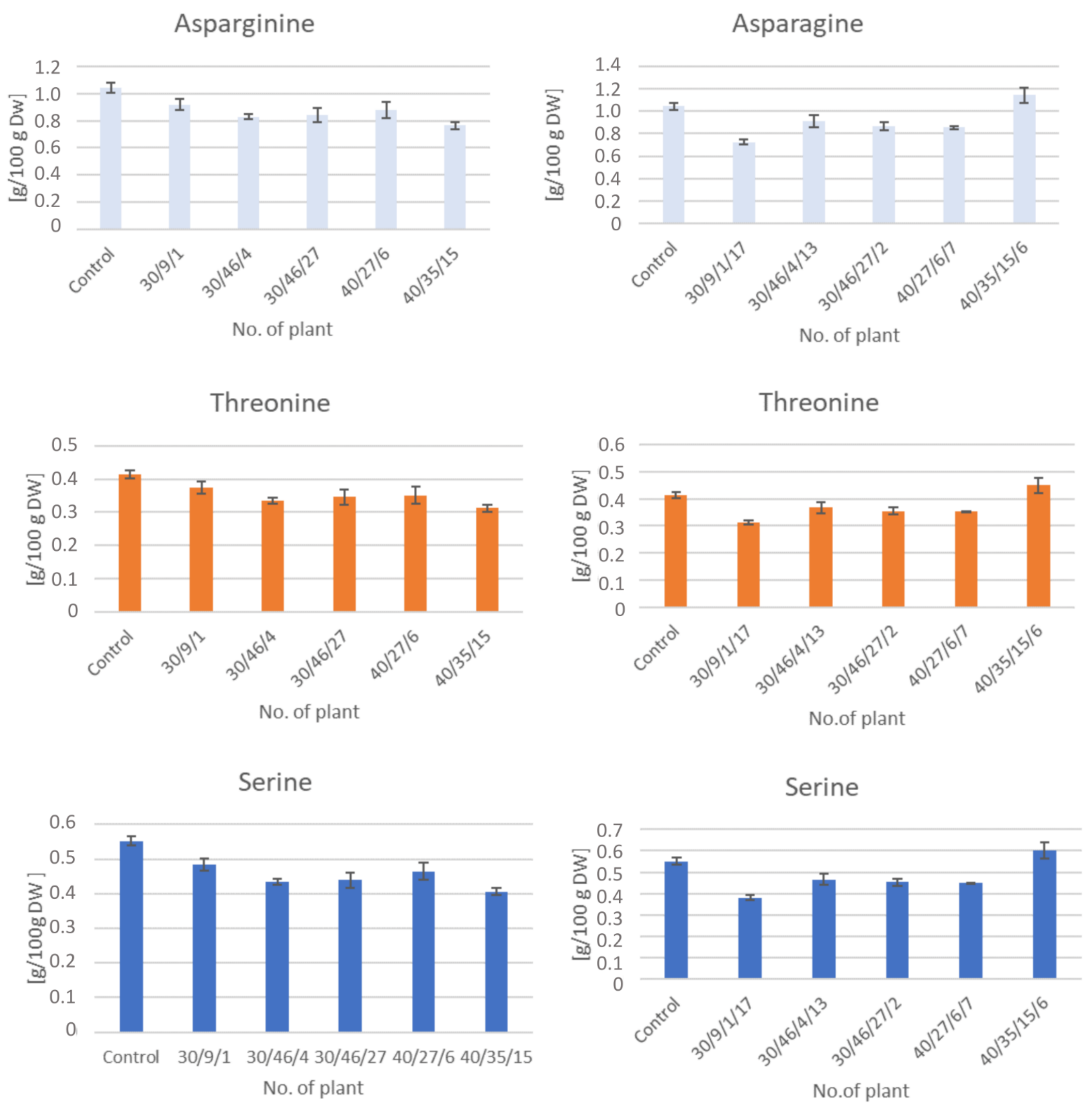

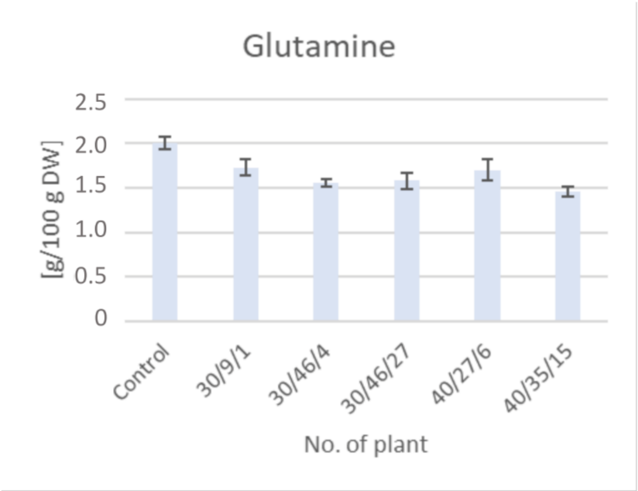
Glutamine
2.5
2.0
1.5
1.0
0.5
0
[g/100 g DW]
Control
30/9/1
30/46/4
30/46/27
40/27/6
40/35/15
No. of plant

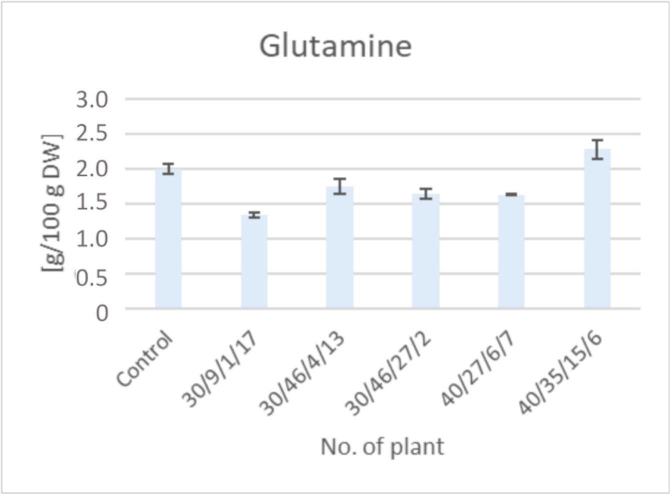
Glutamine
3.0
2.5
2.0
1.5
1.0
0.5
0
[g/100 g DW]
Control
30/9/1/17
30/46/4/13
30/46/27/2
40/27/6/7
40/35/15/6
No. of plant

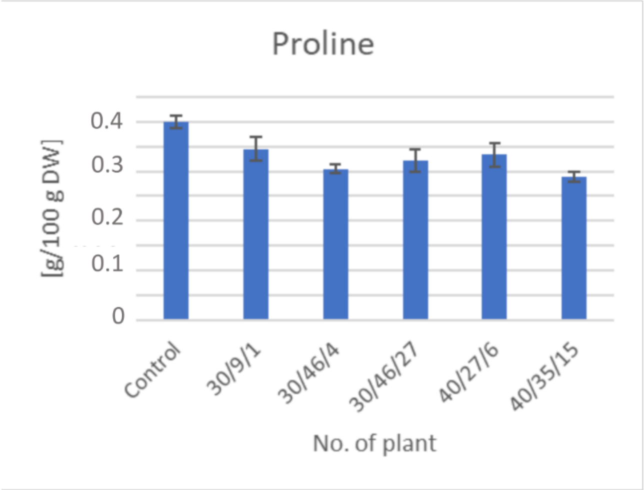
Proline
0.4
0.3
0.2
0.1
0
[g/100 g DW]
Control
30/9/1
30/46/4
30/46/27
40/27/6
40/35/15
No. of plant

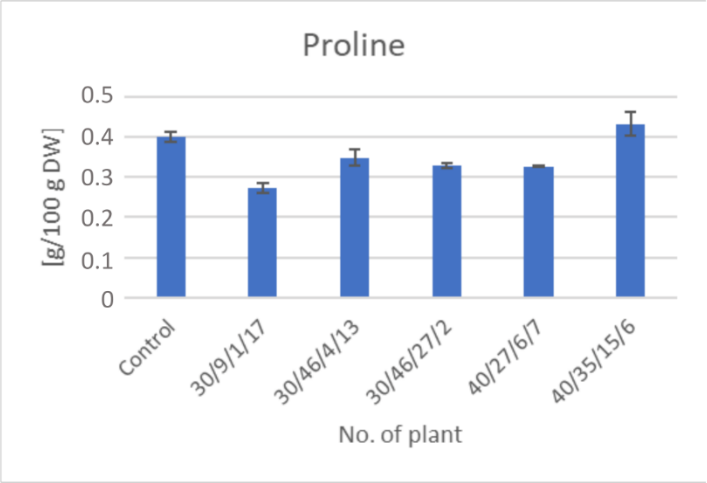
Proline
0.5
0.4
0.3
0.2
0.1
0
[g/100 g DW]
Control
30/9/1/17
30/46/4/13
30/46/27/2
40/27/6/7
40/35/15/6
No. of plant

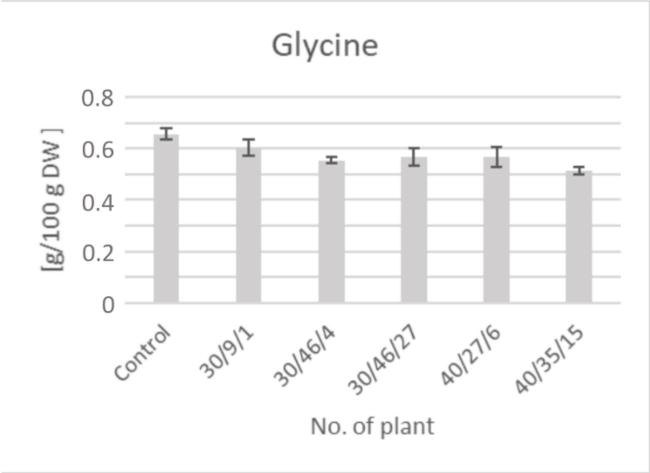
Glycine
0.8
0.6
0.4
0.2
0
[g/100 g DW]
Control
30/9/1
30/46/4
30/46/27
40/27/6
40/35/15
No. of plant

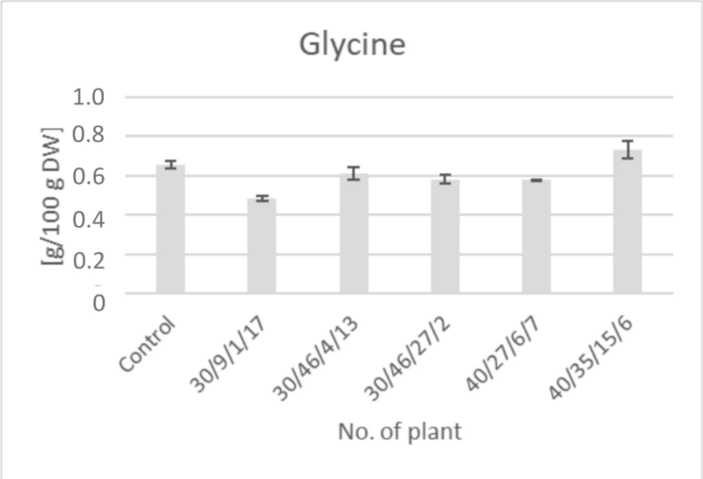
Glycine
1.0
0.8
0.6
0.4
0.2
0
[g/100 g DW]
Control
30/9/1/17
30/46/4/13
30/46/27/2
40/27/6/7
40/35/15/6
No. of plant

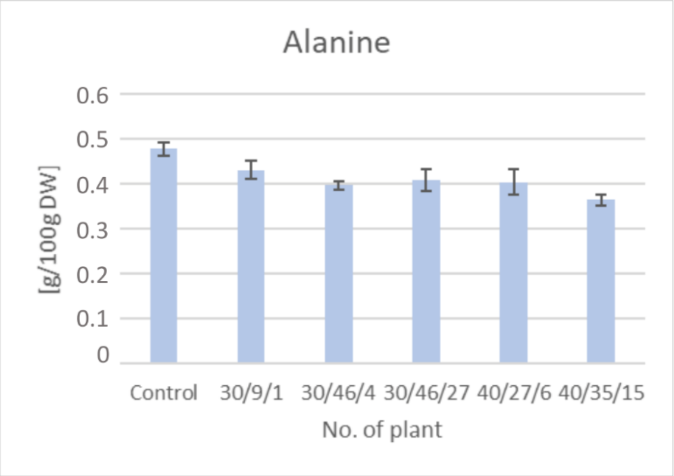
Alanine
0.6
0.5
0.4
0.3
0.2
0.1
0
[g/100g DW]
Control
30/9/1
30/46/4
30/46/27
40/27/6
40/35/15
No. of plant

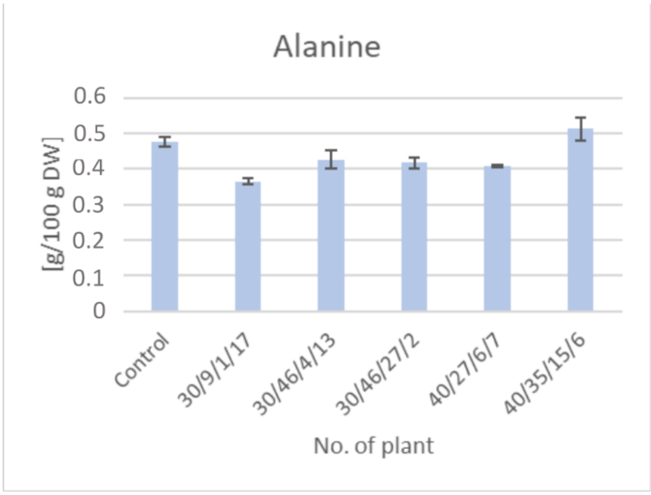
Alanine
0.6
0.5
0.4
0.3
0.2
0.1
0
[g/100 g DW]
Control
30/9/1/17
30/46/4/13
30/46/27/2
40/27/6/7
40/35/15/6
No. of plant

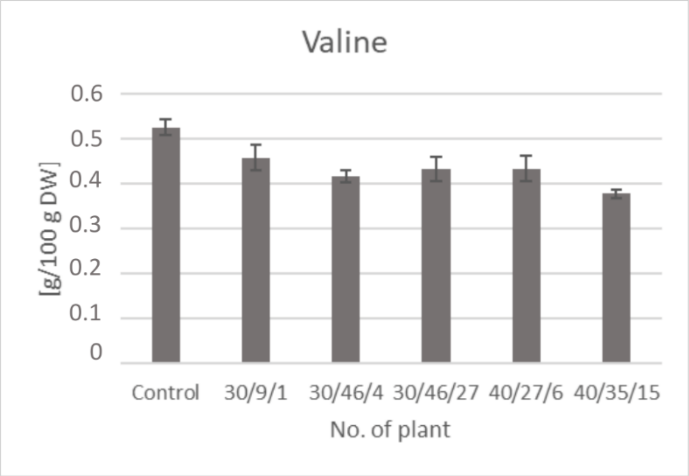
Valine
0.6
0.5
0.4
0.3
0.2
0.1
0
[g/100 g DW]
Control
30/9/1
30/46/4
30/46/27
40/27/6
40/35/15
No. of plant

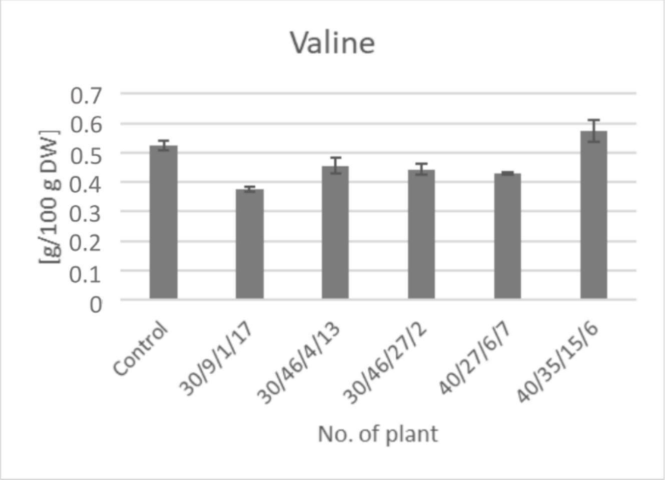
Valine
0.7
0.6
0.5
0.4
0.3
0.2
0.1
0
[g/100 g DW]
Control
30/9/1/17
30/46/4/13
30/46/27/2
40/27/6/7
40/35/15/6
No. of plant

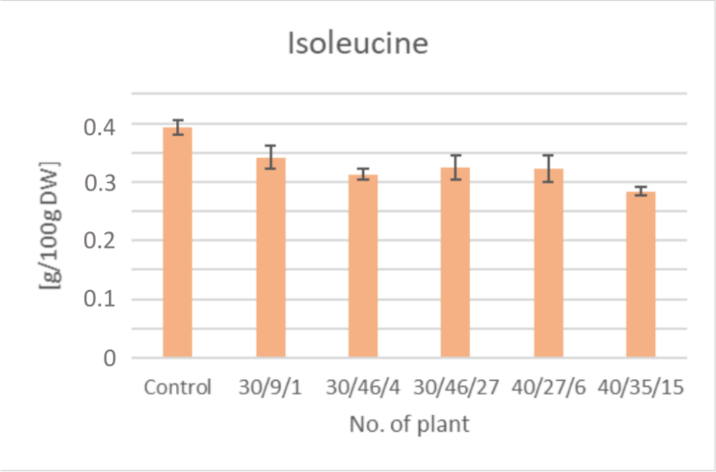
Isoleucine
0.4
0.3
0.2
0.1
0
[g/100g DW]
Control
30/9/1
30/46/4
30/46/27
40/27/6
40/35/15
No. of plant

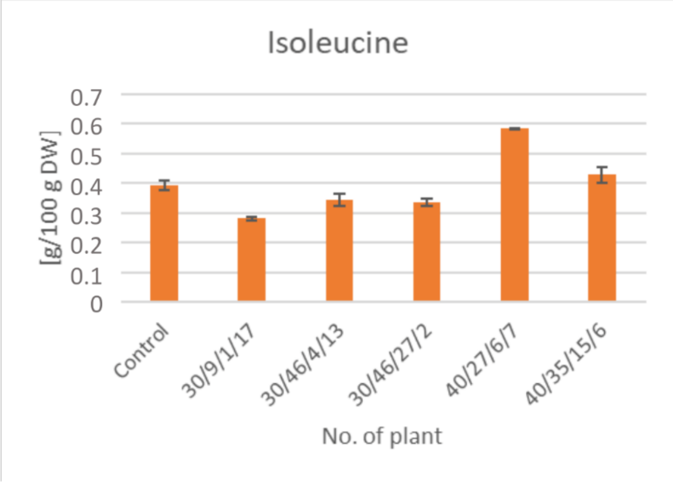
Isoleucine
0.7
0.6
0.5
0.4
0.3
0.2
0.1
0
[g/100 g DW]
Control
30/9/1/17
30/46/4/13
30/46/27/2
40/27/6/7
40/35/15/6
No. of plant

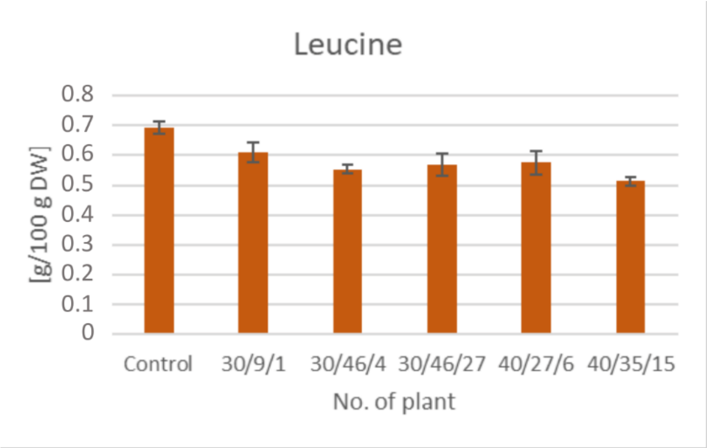
Leucine
[g/100 g DW]
0.8
0.7
0.6
0.5
0.4
0.3
0.2
0.1
0
Control
30/9/1
30/46/4
30/46/27
40/27/6
40/35/15
No. of plant

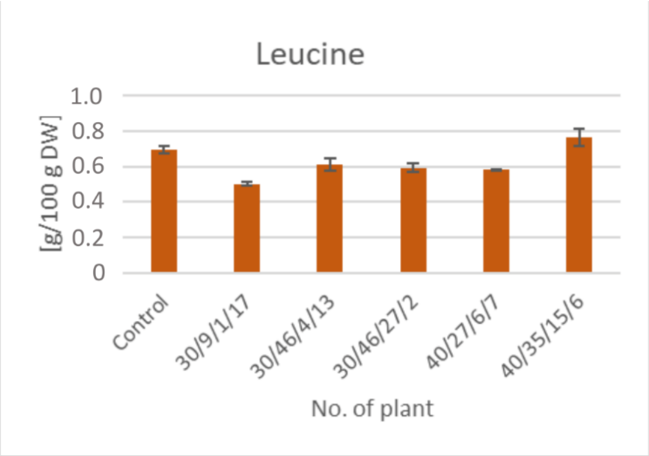
Leucine
[g/100 g DW]
1.0
0.8
0.6
0.4
0.2
0
Control
30/9/1/17
30/46/4/13
30/46/27/2
40/27/6/7
40/35/15/6
No. of plant

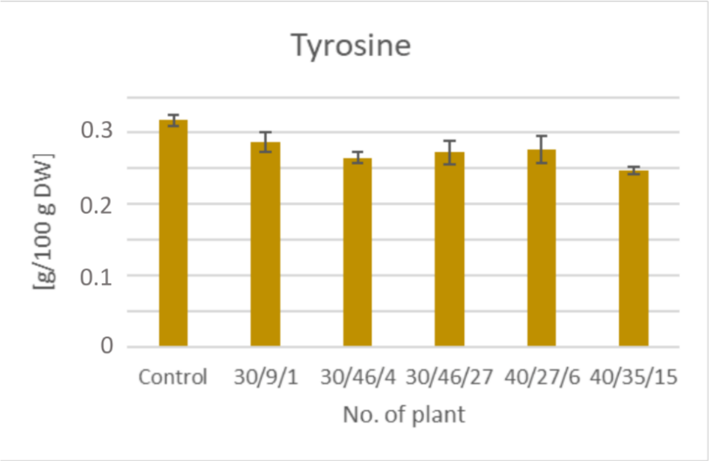
Tyrosine
[g/100 g DW]
0.3
0.2
0.1
0
Control
30/9/1
30/46/4
30/46/27
40/27/6
40/35/15
No. of plant

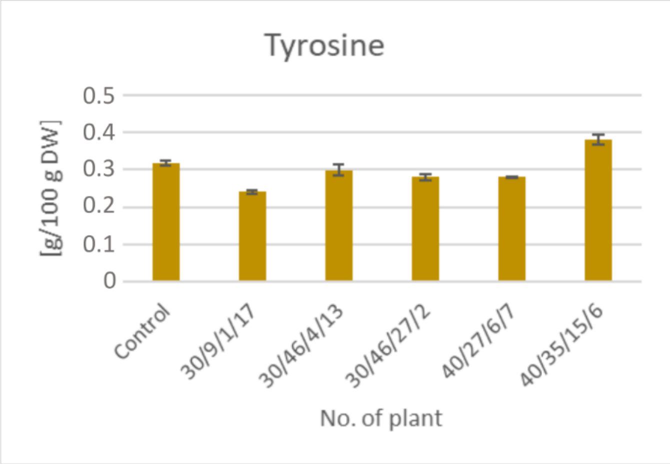
Tyrosine
[g/100 g DW]
0.5
0.4
0.3
0.2
0.1
0
Control
30/9/1/17
30/46/4/13
30/46/27/2
40/27/6/7
40/35/15/6
No. of plant

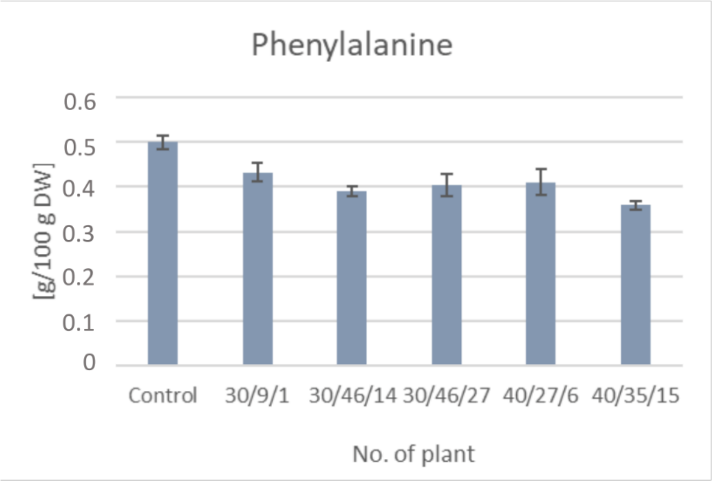
Phenylalanine
[g/100 g DW]
0.6
0.5
0.4
0.3
0.2
0.1
0
Control
30/9/1
30/46/14
30/46/27
40/27/6
40/35/15
No. of plant

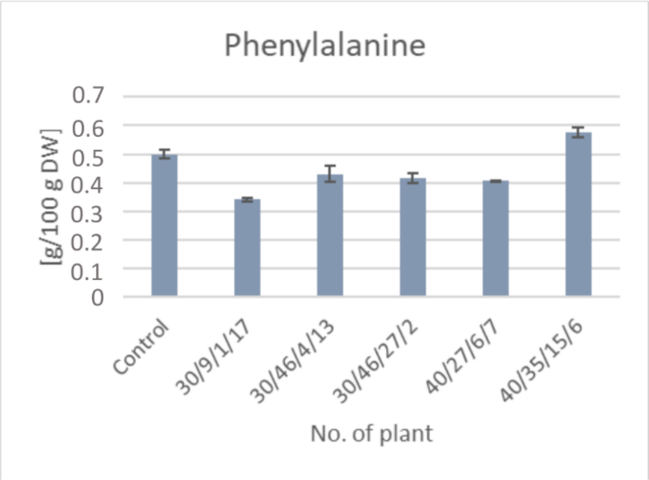
Phenylalanine
[g/100 g DW]
0.7
0.6
0.5
0.4
0.3
0.2
0.1
0
Control
30/9/1/17
30/46/4/13
30/46/27/2
40/27/6/7
40/35/15/6
No. of plant

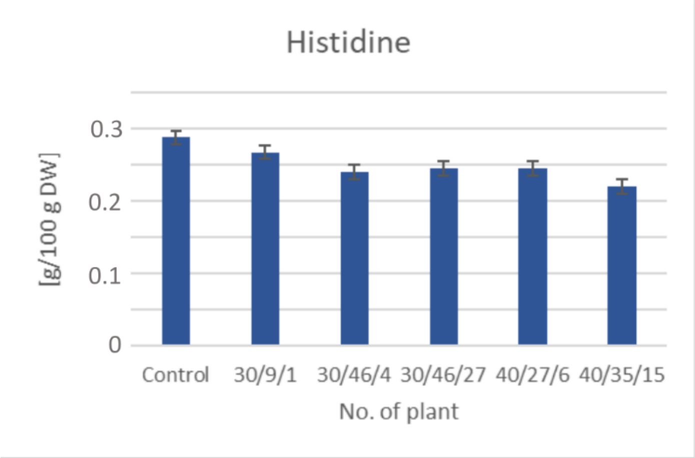
Histidine
0.3
0.2
0.1
0
[g/100 g DW]
Control
30/9/1
30/46/4
30/46/27
40/27/6
40/35/15
No. of plant

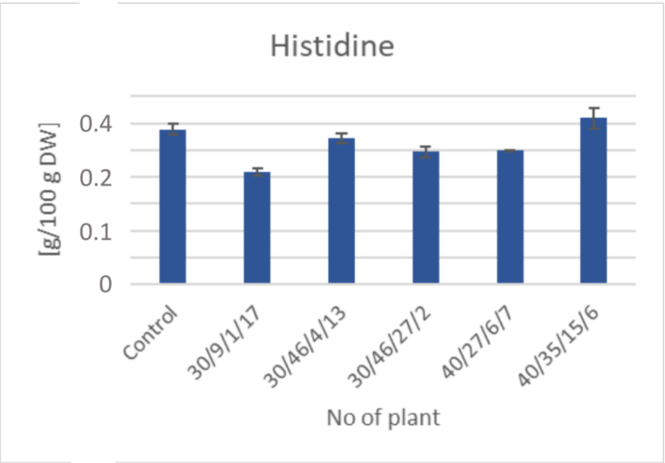
Histidine
0.4
0.2
0.1
0
[g/100 g DW]
Control
30/9/1/17
30/46/4/13
30/46/27/2
40/27/6/7
40/35/15/6
No of plant

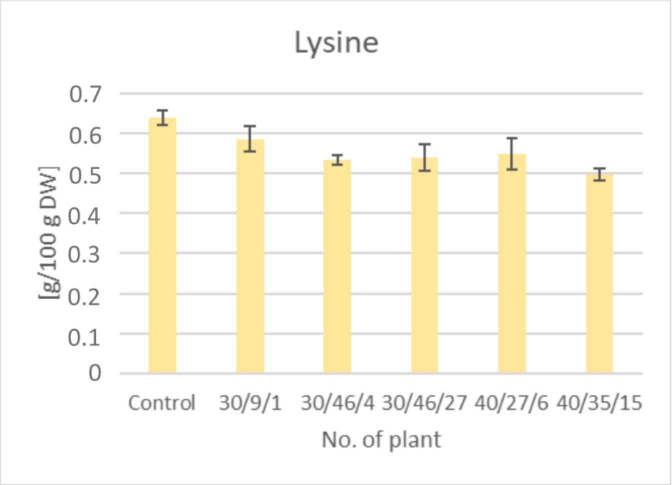
Lysine
0.7
0.6
0.5
0.4
0.3
0.2
0.1
0
[g/100 g DW]
Control
30/9/1
30/46/4
30/46/27
40/27/6
40/35/15
No. of plant

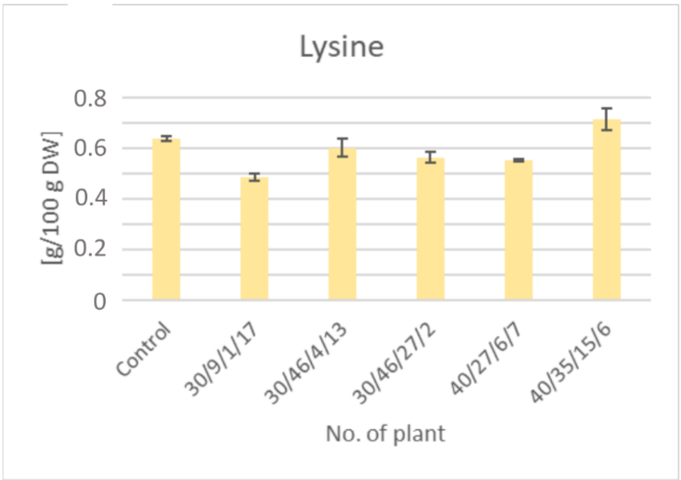
Lysine
0.8
0.6
0.4
0.2
0
[g/100 g DW]
Control
30/9/1/17
30/46/4/13
30/46/27/2
40/27/6/7
40/35/15/6
No. of plant

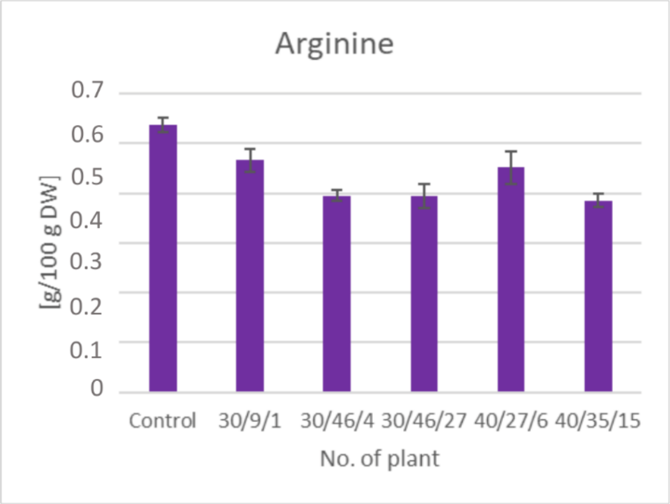
Arginine
0.7
0.6
0.5
0.4
0.3
0.2
0.1
0
[g/100 g DW]
Control
30/9/1
30/46/4
30/46/27
40/27/6
40/35/15
No. of plant

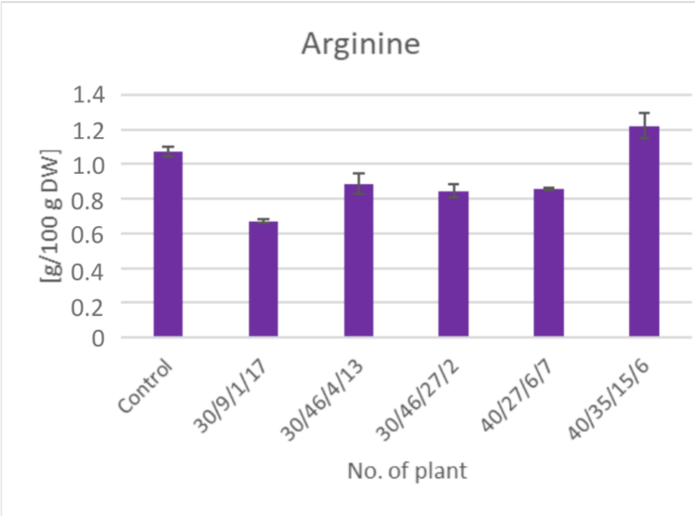
Arginine
1.4
1.2
1.0
0.8
0.6
0.4
0.2
0
[g/100 g DW]
Control
30/9/1/17
30/46/4/13
30/46/27/2
40/27/6/7
40/35/15/6
No. of plant

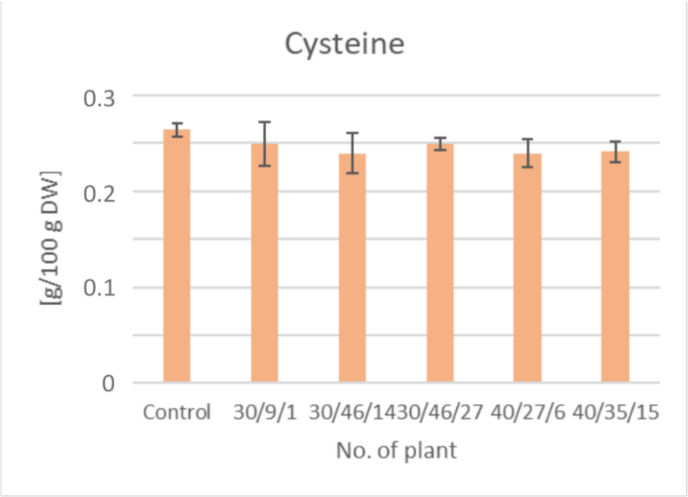
Cysteine
0.3
0.2
0.1
0
[g/100 g DW]
Control
30/9/1
30/46/14
30/46/27
40/27/6
40/35/15
No. of plant

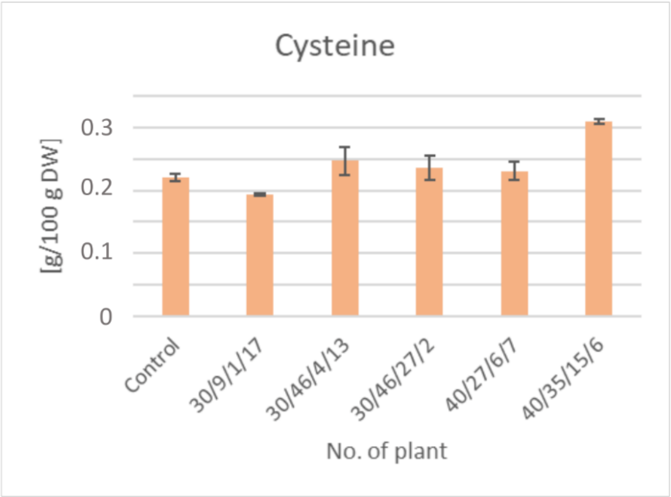
Cysteine
0.3
0.2
0.1
0
[g/100 g DW]
Control
30/9/1/17
30/46/4/13
30/46/27/2
40/27/6/7
40/35/15/6
No. of plant

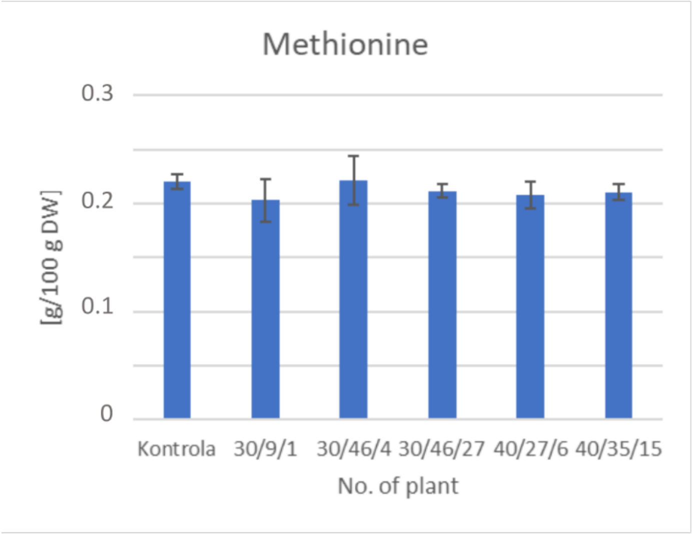
Methionine
0.3
0.2
0.1
0
[g/100 g DW]
Kontrola
30/9/1
30/46/4
30/46/27
40/27/6
40/35/15
No. of plant

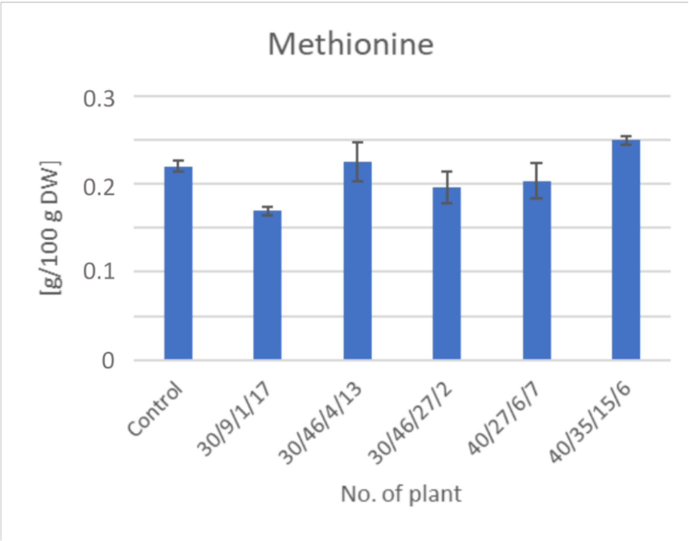
Methionine
0.3
0.2
0.1
0
[g/100 g DW]
Control
30/9/1/17
30/46/4/13
30/46/27/2
40/27/6/7
40/35/15/6
No. of plant
