# Supplementary material for: Higher Yield of Common Buckwheat (Fagopyrum esculentum Moench) as a Result of Seed Treatment with Gamma Radiation
Source: Int J Mol Sci. 2025 May 10;26(10):4587. doi: 10.3390/ijms26104587 (PMC12110951; doi:10.3390/ijms26104587)
Supplement: Supplementary file 1 [file ijms-26-04587-s001.zip › Supplementary Table S1.pdf]

**Supplementary Table S1.** Mean, minimum, maximum and intervals of following parameters of common buckwheat plants grown from seeds irradiated at 30 and 40 Gy (generation M0): number of branches, plant height, fresh (FW) and dry weight (DW) of the aboveground part, number of empty seeds, percentage of empty seeds, number of ripe seeds and their weight, weight of a seed.

| | Control | | | | 30 Gy | | | | 40 Gy | | | |
|---|---|---|---|---|---|---|---|---|---|---|---|---|
| No. plants | 20 | | | | 142 | | | | 75 | | | |
| | Mean | Min. | Max. | Interval | Mean | Min. | Max. | Interval | Mean | Min. | Max. | Interval |
| No. branches | 3.95 | 2 | 6 | 4 | 4.33 | 2 | 10 | 8 | 4.61 | 2 | 9 | 7 |
| High [cm] | 91.85 | 72 | 121 | 49 | 114.20 | 52 | 172 | 120 | 114.2 | 54 | 162 | 108 |
| FW [g] | 54.13 | 35.13 | 101.04 | 65.91 | 60.01 | 24.96 | 123.71 | 98.75 | 67.02 | 26.71 | 174.55 | 147.84 |
| DW [g] | 27.31 | 20.13 | 36.71 | 16.58 | 26.26 | 38.96 | 112.70 | 73.74 | 24.77 | 18.21 | 43.82 | 25.61 |
| No. empty seeds | 21.5 | 4 | 44 | 40 | 48.5 | 0 | 290 | 290 | 37.06 | 0 | 263 | 263 |
| % empty seeds | 11.011 | 1.93 | 27,814 | 36.56 | 21.46 | 0 | 66.98 | 66.98 | 21.61 | 0 | 85.99 | 85.99 |
| No. ripe seeds | 201.6 | 94 | 409 | 315 | 262.9 | 6 | 861 | 855 | 325.1 | 29 | 1027 | 998 |
| Seeds weight [g] | 5.79 | 2.77 | 11.34 | 8.57 | 8.25 | 0.18 | 23.44 | 23.26 | 9.06 | 0.63 | 30.55 | 29.92 |
| 1 seed weight [g] | 0.0303 | 0.0203 | 0.0892 | 0.0689 | 0.0323 | 0.0203 | 0.0992 | 0.0789 | 0.0281 | 0.0190 | 0.0441 | 0.0251 |
