# Supplementary material for: Higher Yield of Common Buckwheat (Fagopyrum esculentum Moench) as a Result of Seed Treatment with Gamma Radiation
Source: Int J Mol Sci. 2025 May 10;26(10):4587. doi: 10.3390/ijms26104587 (PMC12110951; doi:10.3390/ijms26104587)
Supplement: Supplementary file 1 [file ijms-26-04587-s001.zip › Supplementary Table S2.pdf]

Supplementary Table S2. Mean, minimum, maximum and intervals of the following parameters of common buckwheat of M1 generation obtained from plants grown from seeds irradiated at 30 Gy or 40 Gy: number of branches, plant height, number of empty seeds, percentage of empty seeds, number of ripe seeds and their weight, weight of a seed.

	**Control**				**30/9**				**30/33**			
No. plants	20				26				21			
	mean	min	max	interval	mean	min	max	interval	mean	min	max	interval
No. branches	4.6	3	6	3	5.6	4	7	3	3.9	2	6	4
High [cm]	114.47	68	155	87	130.34	95	165	70	14.71	107	182	75
No. empty seeds	74.32	12	194	182	75.65	27	201	174	103.66	35	402	367
% empty seeds	13.5	5.5	27.7	22.2	11.5	5.8	23.7	17.9	20.6	11.0	36.3	25.3
No. ripe seeds	486.5	134	1032	898	576.1	314	1040	726	415.62	145	922	777
Seeds weight [g]	15.46	4.72	30.71	25.99	18.59	10.67	29.11	18.44	13.28	5.61	29.65	24.04
1 seed weight [g]	0.0327	0.0278	0.0496	0.0218	0.0326	0.0280	0.0376	0.0096	0.0328	0.0269	0.0416	0.0147

	Control				30/46				30/131			
No. plants	20				30				19			
	Mean	Min.	Max.	Interval	Mean	Min.	Max.	Interval	Mean	Min.	Max.	Interval
No. branches	4.6	3	6	3	5.6	3	9	6	4.11	2	7	5
High [cm]	114.47	68	155	87	143.2	110	185	75	148.0	115	172	57
No. empty seeds	74.32	12	194	182	48.4	16	139	123	88.2	18	216	198
% empty seeds	13.5	5.5	27.7	22.2	9.9	5.1	19.1	14.0	18.5	5.3	41.9	36.6
No. ripe seeds	486.5	134	1032	898	413.8	158	737	579	380.1	207	715	508
Seeds weight [g]	15.46	4.72	30.71	25.99	14.20	5.54	26.06	20.52	13.89	6.95	27.45	20.40
1 seed weight [g]	0.0327	0.0278	0.0496	0.0218	0.0343	0.0265	0.0481	0.0216	0.0364	0.0304	0.0401	0.0197

	40/11				40/18				40/20			
No. plants	22				22				22			
	Mean	Min.	Max.	Interval	Mean	Min.	Max.	Interval	Mean	Min.	Max.	Interval
No. branches	4.9	3	8	5	5.3	3	7	4	5.5	3	8	5
High [cm]	130.6	83	172	89	137.2	109	167	58	137	103	180	77
No. empty seeds	104.8	0	642	642	72.3	10	232	132	88.8	30	230	200
% empty seeds	15.3	0	34.4	34.4	12.6	2.5	27.1	24.6	17.9	27.2	7.6	19.6
No. ripe seeds	499.4	177	1239	962	460.9	230	1047	817	401.7	161	684	523
Seeds weight [g]	16.28	6.02	35.80	29.78	14.28	7.65	31.39	23.74	12.36	5.12	21.95	16.83
1 seed weight [g]	0.0326	0.0269	0.0381	0.0102	0.0316	0.0264	0.0364	0.0100	0.0311	0.0249	0.0383	0.0134

	**40/23**				**40/27**				**40/35**			
No. plants	7				11				19			
	Mean	Min.	Max.	Interval	Mean	Min.	Max.	Interval	Mean	Min.	Max.	Interval
No. branches	5.9	4	8	4	6	4	7	3	5.5	4	9	5
High [cm]	149.7	130	168	38	146.1	110	171	61	137.3	111	190	79
No. empty seeds	82.4	32	173	151	126.4	60	230	170	36.6	8	100	92
% empty seeds	19.3	7.7	34.3	26.6	21.0	8.8	32.0	23.2	8.7	2.6	17.1	14.5
No. ripe seeds	336.6	158	538	480	478.2	243	901	758	370.1	120	751	631
Seeds weight [g]	10.61	4.36	17.65	13.29	15.30	8.05	32.81	24.76	12.17	6.36	26.80	19.44
1 seed weight [g]	0.0310	0.0275	0.0332	0.0057	0.0315	0.0262	0.0365	0.0103	0.0339	0.0292	0.0615	0.0323
