# Supplementary material for: Higher Yield of Common Buckwheat (Fagopyrum esculentum Moench) as a Result of Seed Treatment with Gamma Radiation
Source: Int J Mol Sci. 2025 May 10;26(10):4587. doi: 10.3390/ijms26104587 (PMC12110951; doi:10.3390/ijms26104587)
Supplement: Supplementary file 1 [file ijms-26-04587-s001.zip › Supplementary Table S3.pdf]

Supplementary Table S3. Mean, minimum, maximum and intervals of following parameters of common buckwheat plants grown from seeds irradiated at 30 or 40 Gy (M2 generation): number of branches, number of empty seeds, percentage of empty seeds, number of ripe seeds and their weight, weight of a seed.

	**Control**				**30/9/1**				**30/46/27**			
No. plants	11				20				9			
	Mean	Min.	Max.	Interval	Mean	Min.	Max.	Interval	Mean	Min.	Max.	Interval
No. branches	3.7	3	4	1	4.9	3	8	5	4.22	3	6	3
High [cm]	135.54	120	176	56	102.8	78	135	57	143.44	114	188	74
No. empty seeds	15.27	1	31	30	39.65	7	103	96	29.9	14	95	81
% empty seeds	4.37	0.38	8.55	8.17	7.104	2.63	23.45	20.82	6.9	2.95	14.26	11.31
No. ripe seeds	310.4	255	389	134	54.04	259	1390	1131	386.89	195	571	376
Seeds weight [g]	12.084	9.142	15.744	6.602	16.68	7.599	44.106	36.507	12.738	7.397	17.282	9.885
1 seed weight [g]	0.0387	0.0366	0.0408	0.0042	0.03104	0.0255	0.0351	0.0096	0.0334	0.0252	0.0379	0.0127

	30/46/4				40/35/15				40/27/6			
No. plants	13				11				9			
	Mean	Min.	Max.	Interval	Mean	Min.	Max.	Interval	Mean	Min.	Max.	Interval
No. branches	4.15	3	6	3	4.8	3	7	4	5.42	4	7	3
High [cm]	140.31	112	171	59	141.9	102	172	70	138	110	173	63
No. empty seeds	43.92	12	121	109	23.9	3	37	34	62.5	14	139	125
% empty seeds	10.14	3.46	21.04	17.58	4.77	0.761	14.684	13.92	76	2.99	23.32	20.33
No. ripe seeds	362	217	571	354	453.6	300	656	356	631.08	289	1042	753
Seeds weight [g]	10.57	5.711	16.326	10.615	13.4	9.366	19.4	10.034	16.957	7.612	23.519	15.907
1 seed weight [g]	0.0289	0.0252	0.0349	0.0097	0.0302	0.0247	0.0366	0.0119	0.0271	0.0221	0.0325	0.0105
